# No cell is an island: characterising the leaf epidermis using epidermalmorph, a new R package

**DOI:** 10.1111/nph.18519

**Published:** 2022-11-15

**Authors:** Matilda J. M. Brown, Gregory J. Jordan

**Affiliations:** ^1^ Biological Sciences University of Tasmania Hobart 7000 Tas. Australia; ^2^ Royal Botanic Gardens Kew Richmond TW9 3AE UK

**Keywords:** cell arrangement, cell shape, epidermal cells, R package, stomatal morphology

## Abstract

- The leaf epidermis is the interface between a plant and its environment. The epidermis is highly variable in morphology, with links to both phylogeny and environment, and this diversity is relevant to several fields, including physiology, functional traits, palaeobotany, taxonomy and developmental biology.
- Describing and measuring leaf epidermal traits remains challenging. Current approaches are either extremely labour‐intensive and not feasible for large studies or limited to measurements of individual cells.
- Here, we present a method to characterise individual cell size, shape (including the effect of neighbouring cells) and arrangement from light microscope images. We provide the first automated characterisation of cell arrangement (from traced images) as well as multiple new shape characteristics. We have implemented this method in an R package, epidermalmorph, and provide an example workflow using this package, which includes functions to evaluate trait reliability and optimal sampling effort for any given group of plants. We demonstrate that our new metrics of cell shape are independent of gross cell shape, unlike existing metrics.
- epidermalmorph provides a broadly applicable method for quantifying epidermal traits that we hope can be used to disentangle the fundamental relationships between form and function in the leaf epidermis.

## Introduction

The leaf epidermis controls transpiration and gas exchange via stomata, protects the plant from both environmental stressors and biological invasions and can even act as a mechano‐sensory organ (Hamant *et al*., 2008). There is extreme diversity in epidermal traits that can be observed using light microscopy – the size, shape, arrangement and numbers of pavement cells, stomatal and other specialised cells (e.g. trichomes) – but the physiological function of each of these traits under different environmental conditions remains unclear, despite extensive study (Sharma, 1972; Royer, 2001; Dunn *et al*., 2015a). The functions of epidermal traits may also vary among groups of plants, highlighting the need for broad‐scale studies of epidermal traits (as noted by Vőfély *et al*., 2019) and thus the need for a widely applicable approach to quantifying and comparing the epidermis.

Leaf epidermal traits are easy to observe in fresh or dried material, and cell imprints are readily preserved via the cuticle, which can be fossilised and thus retain epidermal information for hundreds of millions of years (Blomenkemper *et al*., 2021). This means that we have a record of epidermal traits spanning deep time that can provide a wealth of evolutionary and palaeoecological information. These traits are used widely in the identification of fossil plants (Dilcher, 1974; Hill, 1991; Jordan & Hill, 1999; Deng *et al*., 2017) as well as estimating past climate, vegetation structure and atmospheric carbon dioxide, through either nearest‐living‐relative or physiognomic approaches (Mosbrugger & Utescher, 1997; Jordan, 2011; Dunn *et al*., 2015b). The epidermis has also been studied in developmental (Bidhendi *et al*., 2019) and functional trait contexts (e.g. Osunkoya *et al*., 2014), because some epidermal traits have been linked to life‐history strategies. However, the physiological significance of many epidermal traits is unclear because some traits appear to have contrasting relationships with climate in different groups of plants (Thomas *et al*., 2004; Jordan, 2011; Dunn *et al*., 2015a). It is possible that these contradictory results could be explained by differences in methodology – there are many metrics that can be used to quantify the epidermis, ranging from very simple and intuitive (e.g. cell area) to extremely complicated (e.g. Fourier analysis, Sánchez‐Corrales *et al*., 2018).

Most current methods of quantifying the epidermis fall into one of two categories:

1. High‐throughput measurements of individual cells (e.g. paceqant, Möller *et al*., 2017; gravis, Nowak *et al*., 2021). These methods employ sophisticated algorithms to describe cell shape, but do not either consider the effect of neighbouring cells or measure the arrangement of cells. Some of these methods have been shown to accurately distinguish subtle differences in the cell shape between genotypes (Nowak *et al*., 2021), but the results can be difficult to interpret in terms of functional traits.
2. Manual description of epidermal measurements (e.g. Stark Schilling & Mill, 2011). These methods can describe a wider range of traits (e.g. how cells are arranged and stomatal spacing) but are slow, labour‐intensive and suffer from subjectivity in terms of choice of measurements. The low‐throughput nature of these methods results in significant compromises between sample size of both taxa and cells, which limits the scope of interpretations that can be made from such studies.

Furthermore, both types of approaches tend to suffer from a common limitation – cell shape is measured on individual cells, without considering the effect of neighbours. The shape of an individual epidermal cell cannot be disentangled from that of its neighbours – adjoining cells share a cell wall and must fit together with no spaces between cells. Stomata and underlying venation also influence the shapes of cells (Vőfély *et al*., 2019), so we propose that (1) the epidermis should be measured as a mosaic of connected cells and (2) measurement of epidermal cells should include metrics of both cell shape and spatial relationships among cells. It is also unclear how many epidermal cells are needed to make reliable estimates of trait values for an individual. Most authors take the mean value for 25–30 cells (Carins Murphy *et al*., 2016; Vőfély *et al*., 2019), though some values are based on as few as seven cells (Bush *et al*., 2017), which is unlikely to be a reliable estimate for species, or even individual plants (Clugston *et al*., 2017).

Here, we present a new R package ‘epidermalmorph’, which contains a wide range of descriptors of individual cells including area, aspect ratio and angle, as well as several novel shape descriptors, measures of cell arrangement and functions to optimise sampling effort. We also compare some of these descriptors to those from other algorithms and provide an example workflow for using epidermalmorph.

## Description

In this section, we summarise the functions and traits measured in epidermalmorph. It is not intended to be an exhaustive list of options and arguments in the package – this information is provided in the help pages (e.g. ?extract.epidermal.traits) in the vignettes, and a full reference table of traits is available in Table S1.

**Fig. 1:**
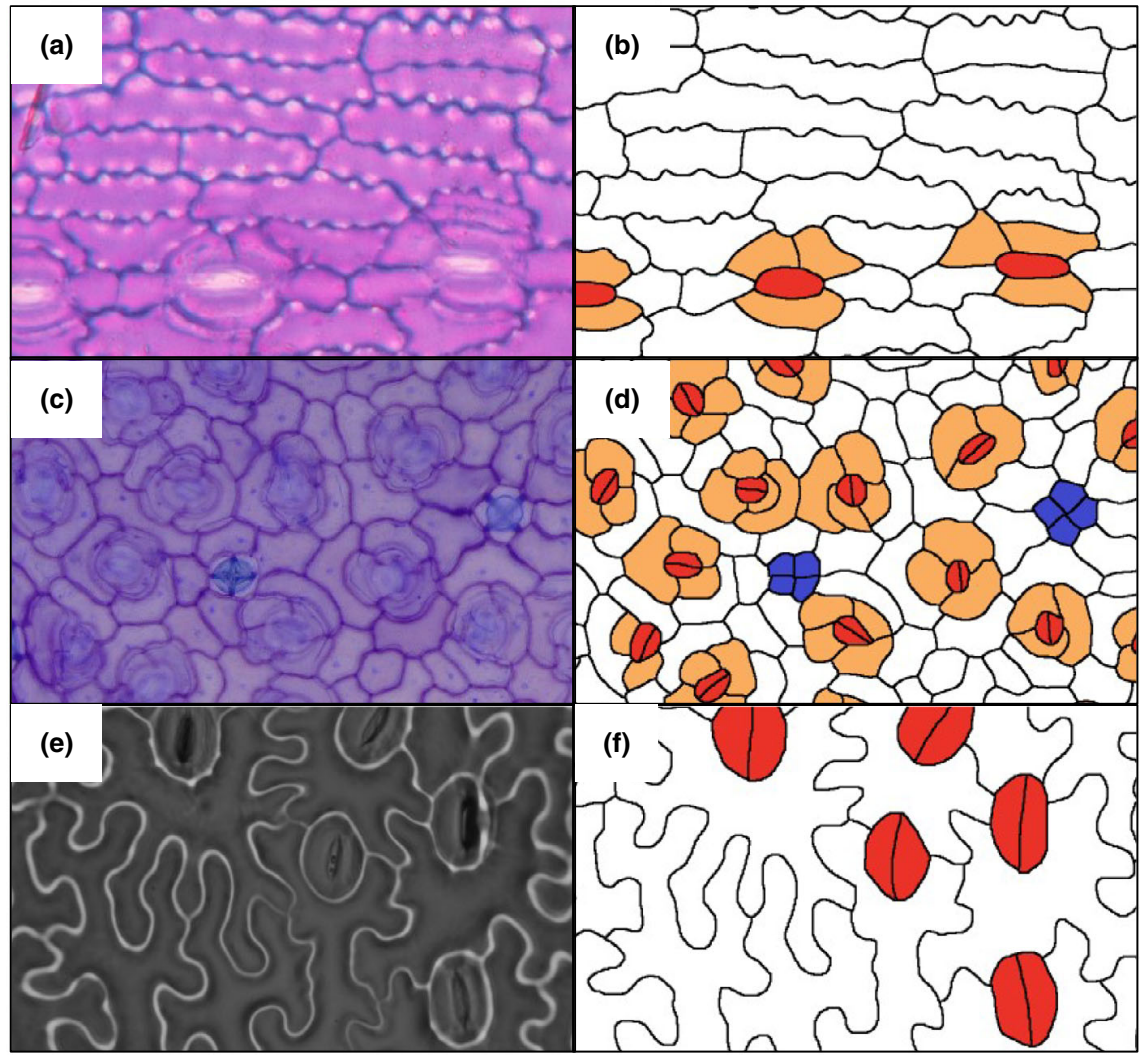
Example images of epidermal cells prepared in various ways and at various resolutions (a, c, e) and the input required for epidermalmorph (b, d, f). The cell walls are shown in black, the pavement cells in white, stomata in red, subsidiary cells in orange and salt glands in blue. Each cell wall is shared between two cells, and there are no empty spaces in the mosaic (apart from the stomatal pores, included with the guard cell pair in (a)). Here, we have used colour to show the different cell types more clearly; for analysis, input values should be a single value between 0 and 255. Images have also been cropped for clarity. *Podocarpus coriaceus* (a, b), light micrograph prepared by authors. *Ceratostigma plumbaginoides* (c, d) and *Lygodium microphyllum* (e, f) undescribed preparation from Vőfély *et al*. (2019). Scales and resolutions vary and have been omitted for visual clarity. Extracted data from these images can be found in the vignette ‘Measuring different epidermal morphologies with epidermalmorph’.

The input image format should be a tracing of the cells such that the cell walls, interiors of pavement cells, stomata and subsidiary cells are each represented by a single value (colour; Fig. 1). Cells can be traced from any image, regardless of magnification, resolution, dimensions or imaging method – the only requirement is that the positions of the cell walls can be reliably identified, either manually or using automated segmentation. Our package does not include an algorithm to automate image tracing, but we recommend using the Ridge Detection function in ImageJ (Steger, 1998; Thorsten Wagner, 2017) or pixel classification in Ilastik (Berg *et al*., 2019) as a form of semiautomation. Tracings produced by these methods normally need to be manually edited before use, though this depends on the method and the image quality; in this study, we found that manual editing added between 10 min and 2 h per image (see the Discussion section and Methods S1).

epidermalmorph also includes a preprocessing function to coerce images into the appropriate form (‘image_preprocess’). The group of pixels for each cell is then converted into a single, spatial polygon object using the user‐provided scale (if not provided, all scale‐dependent measurements are in pixels; see Table S1).

**Fig. 2:**
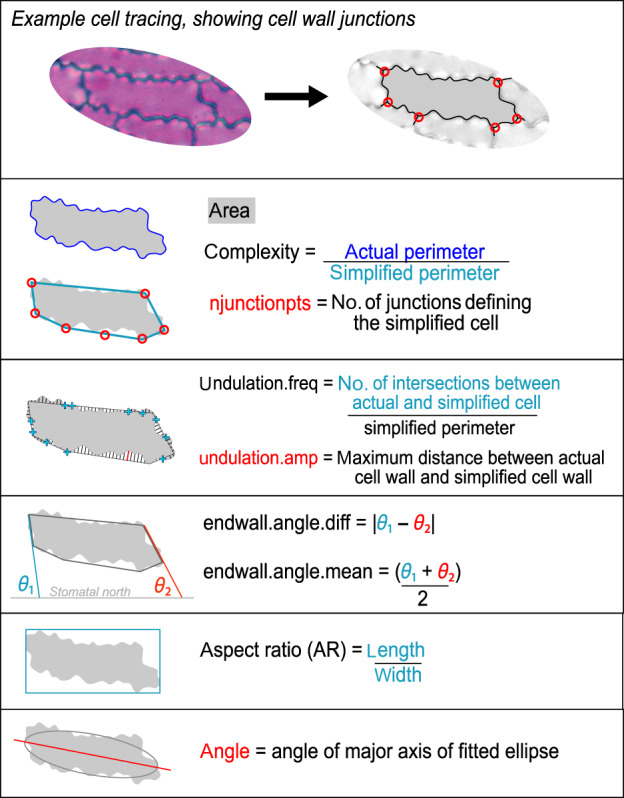
Graphical description of individual pavement cell metrics that can be measured using epidermalmorph.

**Fig. 3:**
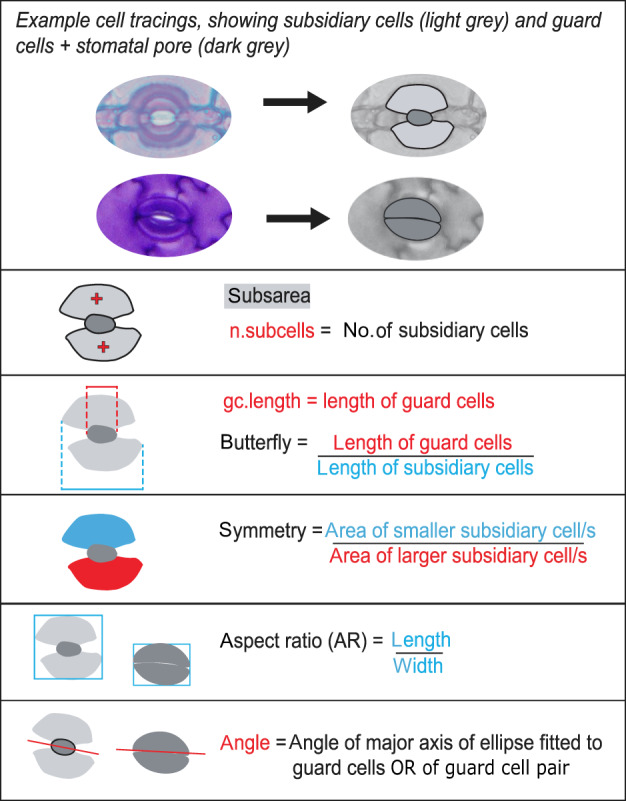
Graphical description of individual stomatal metrics that can be measured using epidermalmorph.

**Fig. 4:**
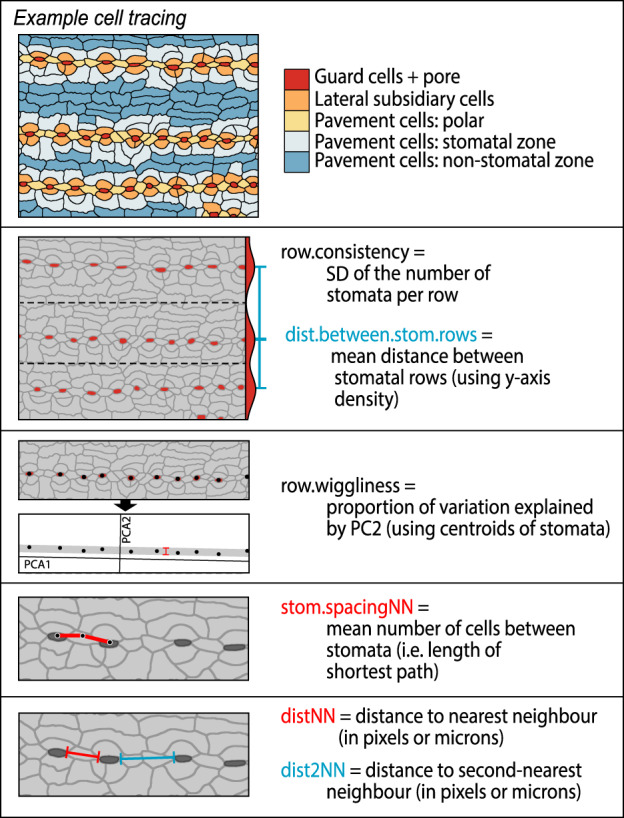
Graphical description of the cell arrangement metrics that can be measured by epidermalmorph. Pavement cells are categorised as being in the ‘pavement zone’, ‘stomatal zone’ or ‘polar’, depending on how far they are from the nearest guard cells. Stomatal arrangement is measured in terms of both row characteristics and spacing. The calculation of ‘row.wiggliness’ is a measure of the deviation of cell centroids from a straight line. This metric is not affected by cell size or spacing; see Supporting Information Table S1 and package documentation for details.

At the core of the epidermalmorph package is the automated measurement of epidermal traits, using the ‘extract_epidermal_traits’ function. These traits include metrics of size, shape and alignment of epidermal cells (Fig. 2) and stomata (Fig. 3), as well as measurements of cell arrangement (Fig. 4). Neighbouring cells are identified, so that the shortest paths between cell types can be calculated (e.g. mean number of cells between stomata, Fig. 4). Cells on the edge of the image are excluded from shape measurements, but cells on the top and left edges are counted to calculate stomatal index (as per Kubínová, 1994).

Stomata can be passed as input to epidermalmorph in two forms. The default is as a single polygon representing the guard cells + pore, similar to the ‘internal stomatal apparatus’ described by Stark Schilling & Mill (2011; see Fig. 1a,b for example). This avoids many problems associated with measuring sunken stomata, where guard cells are obscured by subsidiary cells or lie in a different plane to the rest of the epidermis. However, individual guard cells can be observed in many species (Fig. 1c–f), so we have also included the option to pass individual guard cells, using the ‘paired.guard.cells’ argument (see vignette for practical example). We have not included specific pore measurements in the package, but stomatal pores could be measured as an additional cell type.

epidermalmorph also includes some functionality for additional cell types (e.g. trichomes, oil or salt glands, Fig. 1c,d) (see vignette ‘Measuring different epidermal morphologies with epidermalmorph’ for example). We have also included an ‘NA.value’ argument that excludes sections of images that are not to be measured (e.g. because of damage, blur or obstruction). This argument can also be used for incomplete fields of view, or where cells on the edge of the leaf are to be measured.

Because the orientation of the image relative to the original leaf may be uncertain, we used the mean angle of the stomata as a proxy for leaf axis (in many plants, stomata are arranged approximately parallel to the leaf axis). To identify the angle of each stomate, we fit ellipses to each polygon (using the Fitzgibbon–Pilu–Fisher method, as implemented in the R package ‘conicfit’; Fitzgibbon *et al*., 1999; Gama, 2015) and then extract the angle of the long axis. For paired guard cells, the angle of the dividing line between the two cells is used (see Fig. 3 for illustration). Stomatal north is calculated as the mean stomatal angle. The angles of pavement cells and individual stomata are then calculated relative to stomatal north. This can be disabled if the leaf axis is known or stomata are randomly aligned (see package documentation). Other measurements for which epidermalmorph provides novel automation are the arrangement and spacing of stomata and subsidiary cells (Fig. 4).

For shape measurements, the pixelated outlines of each cell are smoothed using the smoothr package (Strimas‐Mackey, 2021) and the cell junctions are used to define the simplified shape of the cell (Fig. 2). This novel way of describing the shape of the cell has advantages over existing methods (see Comparison to other approaches). Where other methods try to exclude cell junctions, we use them to disentangle gross cell shape from wall undulation. The simplified cell shape is used as a basis for computing three main measures of undulation (Fig. 2): the number of times that the cell wall crosses the simplified perimeter per millimetre (‘undulation.freq’, analogous to frequency), the maximum distance between the cell wall and the simplified perimeter (‘undulation.amp’; analogous to amplitude), and the ratio between the minimum perimeter and actual perimeter (‘complexity’).

We implemented epidermalmorph in R, because it is a program widely used by biologists, and we wished to make this method accessible. The R package is available on GitHub (https://github.com/matildabrown/epidermalmorph).

### Sampling effort

Tracing epidermal cells is time‐consuming, even with partial automation (see the Discussion section), so finding a balance between effort and information is crucial. We have included a workflow to identify optimum sampling effort in the epidermalmorph package that employs subsampling of cells within images.

**Fig. 5:**
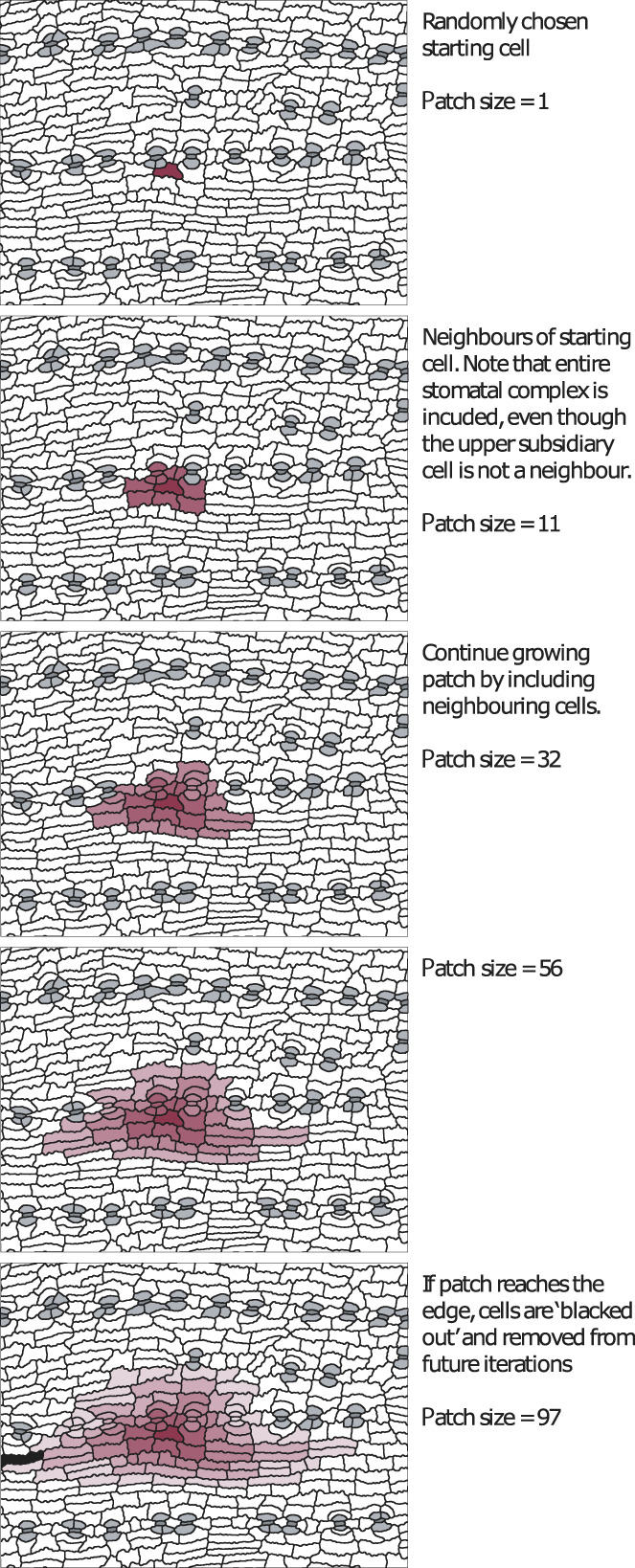
Example of cell patch sampling. From a starting cell, the region is grown iteratively by including neighbouring cells (fading colour intensity is used here to show distance from the starting cell). If part of a stomatal complex is included, the algorithm automatically includes all other cells from that complex. If an edge is reached, the cell that is broken by the edge is removed and not included in future iterations.

For each image, the patch_sampler() function samples and measures cells in random, contiguous patches of varying sizes (e.g. 50, 100, 200 and 400 cells). In this function, a single cell is randomly selected from all nonedge cells, then the neighbours of that cell are added to the patch, then the neighbours of those cells and so forth until the patch exceeds the required size. Where part of a stomatal complex is encountered, all cells from that complex are automatically added to the patch. This process is illustrated in Fig. 5. Although not part of our example workflow, this function might also be used to measure epidermal trait heterogeneity over the leaf surface.

The variance in measured trait values from patches can then be used to identify a minimum number of cells, either by using an arbitrary threshold (e.g. the number of cells required to achieve < 10% variance in samples) or some other measure of convergence (see the package documentation for details). We also recommend carrying out pilot studies to evaluate trait reliability, because some traits measured using epidermalmorph may not be useful for a given study (see ‘Trait reliability’ in the Example workflow section and the Discussion section).

### Comparison with existing methods

To the best of our knowledge, epidermalmorph is the first piece of software that describes the spatial distribution of stomata on the leaf surface. epidermalmorph measures six traits describing the arrangement of stomata (Fig. 4), including the spacing of both individual complexes and rows of stomata, as well as whether these rows are discontinuous (row.consistency) and straight/crooked (row.wiggliness). These traits can be distinctive in certain groups of plants and have been used to identify fossils (Hill & Pole, 1992; Andruchow‐Colombo *et al*., 2019), so we envisage that epidermalmorph may be useful for automated identification of plants from cuticle fragments. Wilf *et al*. (2016) demonstrated that automated identification of leaf fossils has significant potential, but epidermal cells have not yet been utilised in this way – possibly because the individual shapes of fern, angiosperm and conifer cells overlap considerably (despite significant differences in mean trait values; Vőfély *et al*., 2019), making identification at even such a coarse taxonomic resolution impossible. By integrating individual cell measurements with stomatal arrangement traits, we expect to find clearer separation between these groups.

While there is some overlap between epidermalmorph and other ways of quantifying the size and shape of cells (e.g. cell area and aspect ratio), only our method describes the shape in the context of the cell's neighbours. The polygon formed by the cell junctions defines the hypothetical shape of the cell if there were no undulations in the shared wall between cells (here termed the simplified cell; illustrated in Fig. 2). We can then measure certain traits from this simplified cell (aspect ratio and endwall angles), but the key advantage this provides is in measuring the undulation of the cell. The degree and type of undulation of the cell wall is one of the most obvious differences between epidermal cells from different plant groups or environments, and unlike other traits (e.g. cell area), there is no obvious single measurement to describe these differences. As such, numerous approaches to measuring undulation have been proposed (Table 1). Good undulation metrics should have the following properties:

1. A cell with straight cell walls will have the lowest possible value of the metric, regardless of gross cell shape.
2. The metric value/s should increase with both the size and number (or frequency) of undulations and should not be affected by magnification.
3. The metric/s should be applicable to cells with very slight undulations as well as those with very large undulations (e.g. the puzzle‐shaped cells of *Arabidopsis*).
4. Metrics of undulation should be readily interpretable.
5. The metric should be able to be implemented easily across a wide range of platforms without specialised software.
6. The metric should be size‐independent.

**Table 1:** Current measurements of cell undulation and their limitations.

Measurement name	Description	Implemented in/by	Limitations
Undulation index, circularity, form factor	Ratio of cell perimeter compared with the perimeter of a circle with the same area	Thomas *et al*. (2004); Bai *et al*. (2010); Andriankaja *et al*. (2012)	Affected by aspect ratio
Solidity	Ratio of cell area to area of convex hull	Vőfély *et al*. (2019); broadly similar to that used by Dunn *et al*. (2015a)	Affected by nonconvex (e.g. crescent‐shaped) cells
Convexity, lobeyness	Ratio of cell perimeter to perimeter of convex hull	PaCeQuant (Möller *et al*., 2017; Sapala *et al*., 2019)	Affected by nonconvex (e.g. crescent‐shaped) cells, though much less than solidity
Margin roughness	The average angle between points on the cell wall compared with the average angle between the same number of points on a circle	McLellan & Endler (1998); PaCeQuant (Möller *et al*., 2017)	Difficult to implement for large numbers of cells. Does not behave as expected with amplitude and/or frequency
Completeness of visibility graph	Related to the direct ‘lines of sight’ between points on the perimeter of the cell; see Nowak *et al*. (2021) for details.	gravis (Nowak *et al*., 2021)	Likely to be affected by nonconvex cells; downloaded software (gravis, GitHub link) does not work for all cell shapes
Skeleton measurements, lobe measurements	Numerous (15) measurements including average basal lobe width, nonlobe area and average branch length. See supporting information of Möller *et al*. (2017) for details	PaCeQuant (Möller *et al*., 2017)	Large number of metrics not linked to traits, limited applicability outside *Arabidopsis*
Elliptical fourier analysis	Based on the fourier series	Sapala *et al*. (2019)	Good at identifying aspect ratio but describes undulation poorly (Vőfély *et al*., 2019). Cannot deal with cells where the lobes ‘double back’ on themselves (nonholomorphic)
Lobe contribution elliptical fourier analysis (LOCO‐EFA)	Modification of Elliptical fourier analysis; see Sánchez‐Corrales *et al*. (2018) for details	Sánchez‐Corrales *et al*. (2018)	Different shapes use different numbers of *L*_*n*_ metrics, difficult to relate to traits

To identify the limitations of existing metrics and illustrate the advantages of epidermalmorph, we simulated 1200 cells of varying shapes and degrees of undulation (see Fig. S1) and then compared the measured values of undulation. The cells from Vőfély *et al*. (2019; also used by Nowak *et al*., 2021) could not be used to evaluate epidermalmorph, because cell junction coordinates were not captured in that dataset. Most existing methods of describing undulation (Table 1) compare the perimeter or area of the cell to some baseline, usually either the convex hull of the cell or a circle with the same area. Circle‐based measurements are extremely sensitive to changes in the gross cell shape – elongated, straight‐walled cells can have the same undulation index as isodiametric, undulated cells (Fig. S2). Convex hull measurements are affected by nonconvex cells (e.g. boomerang or crescent shape), and none of the single‐value metrics can differentiate between many small undulations and few large undulations (Vőfély *et al*., 2019, though see Fig. S3).

Other measures are indifferent to gross cell shape, but produce a large set of variables that can be difficult to interpret in a trait framework – for example, gravis, a recently developed method that uses visibility graphs to describe the cell (Nowak *et al*., 2021). gravis generates a graph for each cell by placing a number of nodes along the cell wall and then joining pairs of these nodes with edges if they can ‘see’ each other (without being occluded by the cell wall). These edges can then be weighted according to their distance, and cell shape can be described as an *n* × *n* visibility matrix (where *n* is the number of nodes). This can be compared between cells using distance metrics or condensed to a single value (the graph density). gravis performs extremely well when classifying different genotypes or identifying cell lobes of *Arabidopsis* and is able to reproduce cell shapes, but cannot be applied to all of our simulated cells and so may be of limited utility for analysing broad‐scale relationships between form and function.

Another relatively recent method is PaCeQuant (Pavement Cell Quantifier), developed by Möller *et al*. (2017) and implemented as a plugin through ImageJ. PaCeQuant quantifies 27 cell shape variables, including skeleton‐based and contour‐based measurements. Cell undulation is captured by circularity, solidity, convexity and margin roughness (Table 1). Margin roughness (described by McLellan & Endler, 1998) represents an alternative approach to measuring undulation because it compares the *angles* between points to the expected angle if they were on a circle (rather than area or perimeter). PaCeQuant has largely been used to study *Arabidopsis* cells, possibly because many of the features measured are specific to the extreme lobing displayed by the model species.

Our method separates gross cell shape (e.g. aspect ratio) from undulation by using the junction points with neighbouring cells to define the shape of the cell if all cell walls were straight (the simplified cell). From this, we introduce the ‘complexity’ metric – the ratio of the cell perimeter to the perimeter of the simplified cell. This is conceptually similar to undulation index, convexity and lobeyness but is not affected by the underlying shape of the cell (Fig. S4). To further characterise the pattern of undulation, we also introduce two additional new measurements: maximum undulation amplitude (undulation.amp; Figs 2, S5) and undulation frequency (undulation.freq, Figs 2, S6). A few, large undulations will be represented as high maximum undulation amplitude, but low undulation frequency, while the opposite will be true for cells with many small undulations. These measurements are approximately analogous to the ‘pitch’ and ‘amplitude’ of Sánchez‐Corrales *et al*. (2018).

On our simulated cells, we found that existing methods performed poorly on elongated or nonconvex cells (Figs S2, S3), but that the metrics from epidermalmorph were not affected (Figs S4–S6). As a single metric, complexity fulfils all of the requirements for a good undulation metric, but the addition of undulation amplitude and frequency improve the characterisation of undulation patterning.

Other approaches to characterising cellular geometry include MorphographX (four‐dimensional input data; de Reuille *et al*., 2015) and recent work by Xie *et al*. (2021). While some measurements are similar between methods (e.g. cell area and guard cell length), they may be calculated differently. For example, Xie *et al*. (2021) calculate stomatal dimensions from the fitted ellipse, rather than from the cells directly (as in epidermalmorph). To the best of the authors' knowledge, epidermalmorph is also the only software that treats pavement cells according to their proximity to (and thus distortion by) stomatal complexes. Traits not included in epidermalmorph (e.g. total pavement cell area; Xie *et al*., 2021) can be calculated from the polygons directly.

## Example workflow

### Example image dataset: Podocarpaceae

To demonstrate the implementation of epidermalmorph, here we present an example workflow using the Podocarpaceae, a conifer family with a largely southern hemisphere distribution comprising 20 genera and 196 species (Farjon, 2010; Page, 2019). Podocarps occupy a wide range of environments from alpine heath (e.g. *Podocarpus lawrencei*) and tropical forest (e.g. *Acmopyle pancheri*) to understorey shrubs in fire‐prone environments (e.g. *P. drouynianus*). They also have strong fossil records (Hill & Brodribb, 1999), and several epidermal studies have been carried out (Wells & Hill, 1989; Stark Schilling & Mill, 2011; Clugston *et al*., 2017), so it is possible to verify that our interpretation of light photomicrographs is consistent with images captured using a scanning electron microscope. Examples for other species (including those illustrated in Fig. 1) are given in the package vignette.

**Fig. 6:**
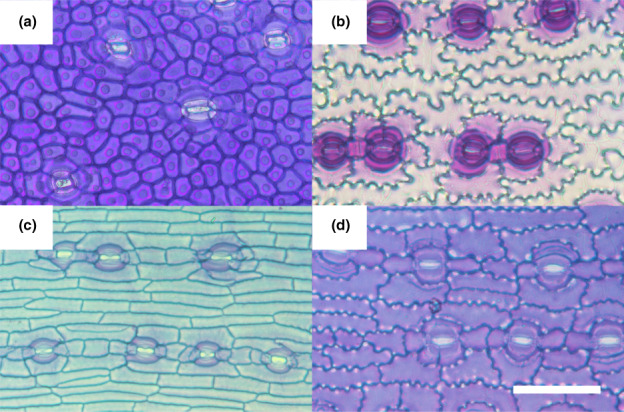
Epidermal diversity in Podocarpaceae. *Phyllocladus aspleniifolius* (a), *Sundacarpus amarus* (b), *Afrocarpus gracilior* (c) and *Podocarpus costalis* (d). Bar, 0.1 mm; same scale for all images.

Epidermal characters vary significantly within Podocarpaceae (Fig. 6). Although stomata generally have two lateral subsidiary cells in a paracytic arrangement, extra divisions often result in additional subsidiary cells. Polar subsidiary cells may be present or absent, and the epidermal cell wall may be straight or strongly sinuous. Gross pavement cell shapes range from more or less isodiametric (e.g. *Acmopyle*), to rectangular (e.g. *P. dispermus*) to irregular (e.g. *P. drouynianus*). Epidermal traits are known to vary between species in the Podocarpaceae (e.g. Wells & Hill, 1989; Stark Schilling & Mill, 2011), and there are well‐resolved phylogenies of this family (Biffin *et al*., 2011; Leslie *et al*., 2018).

### Image preparation

We collected, prepared and imaged cuticles from 26 individual glasshouse‐grown plants (spanning 20 species and seven genera, see Table S2; Methods S1 for details). For each plant, we captured between three and five images from two to four leaves, and they were then traced using a combination of the Ridge Detection plugin from ImageJ (based on the method described by Steger, 1998) and manual tracing. Stomata and subsidiary cells were manually annotated using a flood fill (using different values for different cell types; see Fig. 1 for example).

### Trait reliability

**Fig. 7:**
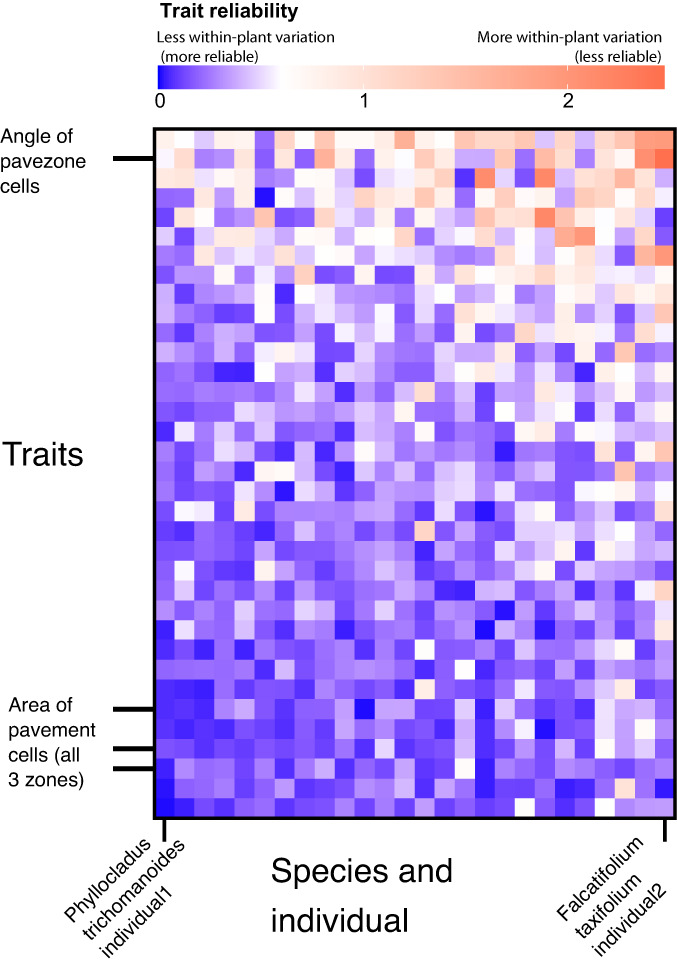
Within‐plant trait reliability scores. The reliability score is calculated as the SD of the standardised trait values measured from the same plant. A reliability score of 1 means that the variation in the plant is equal to the variation across plants, a score of 3 means that the within‐plant variation is three times higher than that of the whole dataset (red, unreliable), while a reliability score of 0.2 means that the within‐plant variation is 20% of the variation across plants (blue, reliable). In this dataset, the traits at the top of the figure (e.g. angle of pavezone cells; see Figs 2, 3 for illustration) are the least reliable (i.e. most variable) when averaged across all plants. Similarly, the plants towards the right of the figure tend to be less reliable (*Falcatifolium taxoides* 2). A fully labelled version of this figure is available in Supporting Information Fig. S7.

We then used epidermalmorph to convert these images to polygons using the ‘image_to_poly’ function and extracted values for all measurements using the ‘extract_epidermal_traits’ function. We scaled and centred these measurements using a *z*‐transformation and then calculated the mean within‐plant standard deviation for each trait using the ‘epidermal_trait_reliability’ function (Fig. 7). We removed all variables that had a mean within‐plant SD > 0.2 (i.e. where the average within‐plant SD exceeded 20% of the total SD for that trait).

**Fig. 8:**
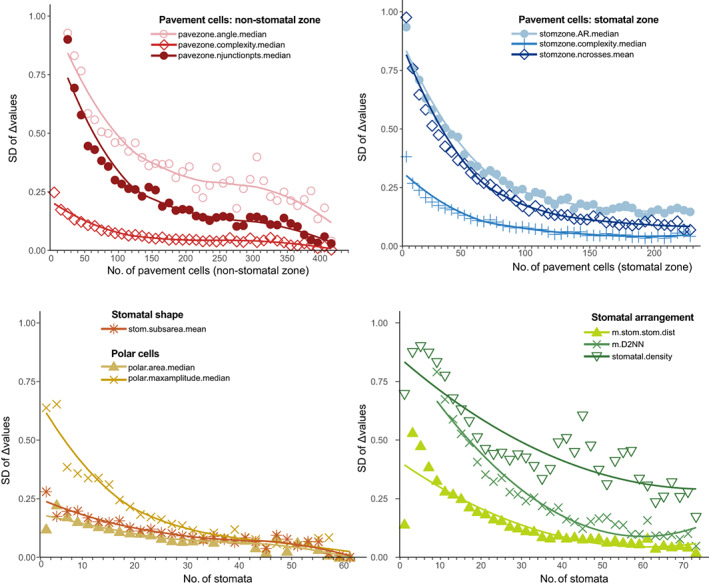
Convergence of measurements taken from subsamples of images. Cells from each image were sampled 100 times at minimum patch sizes of 50, 100, 200 and 400 cells. Traits from each of these samples were measured, and the difference between the sample and the whole image was calculated for each trait (the Δvalues). We then calculated the SD of the Δvalues to evaluate convergence, to determine the optimal number of cells to sample. Lines were fitted using ‘stat_smooth’ in ggplot2 (Wickham, 2016). From this plot, we can determine the variability between patch sizes, and by choosing an appropriate threshold (e.g. 0.1), we can find the minimum number of cells required to measure each trait. In this example, we have included some ‘unreliable’ traits (e.g. stomatal density, where the SD of the Δvalues remains above 0.3 even for maximum patch sizes) – see the package vignette for an example where traits are filtered by reliability before this step.

To calculate optimum sampling effort, we re‐measured our metrics on subregions of each image using the ‘subsample_epidermal_traits’ function. Measurements were extracted from each patch as for the whole image and scaled using the mean and SDs of the trait across the whole‐image dataset. We repeated this 100 times for each image at each minimum patch size (50, 100, 200 and 400 cells) and recorded the number of cells of each cell type (pavement, stomata and subsidiary) for each iteration. We then calculated the difference between the patch and whole‐image value (the delta value) to allow us to compare measurement error between images. From this, we calculated the SD of the delta values (see Fig. 8) at various patch sizes. Low SDs of delta values indicate that all patch measurements were similar to each other (i.e. convergent), and high values indicate that the measurements varied depending on which cells were included in the patch (i.e. the patch size was too small to provide a consistent measurement). For Podocarpaceae, 100–200 pavement cells and 30–40 stomata are required to get a reliable estimate of most trait values (Fig. 8). We provide a function that runs these steps to summarise the output of ‘subsample_epidermal_traits’ and generates a summary data.frame as well as optional plots (‘subsampling_summary’; see package vignette and documentation).

## Discussion

epidermalmorph is the first software to synthesise the low‐throughput measurements used by palaeobotanists and the quantitative, big‐data approach that has been widely adopted to study individual cell traits. Our new metrics of cell wall undulation are unaffected by gross cell shape and can be easily interpreted in a functional trait context. Our method is open‐source, modular and can be applied to a wide range of plants, which is vital to understanding the functional significance of leaf epidermal traits.

Part of the problem with untangling the functionality of epidermal traits seems to be that traits are under the control of different factors in different groups of plants. Some traits are controlled by evolutionary history (e.g. monocot stomatal shape), and there is evidence of phylogenetic signal in pavement cell shape (Vőfély *et al*., 2019), but environment can also have significant direct effects on epidermal traits – one example of this is the difference between sun and shade leaves of the same individual (e.g. Bruschi *et al*., 2000). Another widely utilised trait–environment relationship is the correlation between stomatal index and atmospheric carbon dioxide concentration in fossils of various groups of plants (McElwain *et al*., 2016), though this is not without controversy (Jordan, 2011).

It may be that trait–climate relationships are not simple and universal because they are clade‐specific. Undulation in the periclinal walls of pavement cells is a prime example; it has been linked to light environment in many angiosperms (Metcalfe & Chalk, 1979; Kürschner, 1997; Thomas *et al*., 2004), but not in grasses (Dunn *et al*., 2015a). Furthermore, some species have deeply lobed cells regardless of environment (e.g. *Arabidopsis*), while others show significant plasticity in this trait (e.g. *Quercus*; Bruschi *et al*., 2000). Several functions of undulation have been hypothesised, including increased support for larger cells (Sapala *et al*., 2019), leaf flexibility (Sotiriou *et al*., 2018), biomechanical integrity (Jacques *et al*., 2014) and increased surface area for cell–cell transport (Galletti & Ingram, 2015), but none of these explanations explains undulation at a broad phylogenetic scale (Vőfély *et al*., 2019). Cell elongation (aspect ratio) is another example – it can be used to predict the leaf aspect ratio (length to width) in monocots, ferns and gymnosperms, but not in angiosperms (Vőfély *et al*., 2019). These findings demonstrate the limitations of generalising trait–function relationships in closely related species and the need to account for phylogenetic scope and structure in analyses of epidermal traits, something that we hope epidermalmorph will facilitate.

The quality of the output of epidermalmorph is dependent on the accuracy of the input segmentation, which therefore depends on the character and quality of the original image. Although we have made every effort to reduce the impact of tracing errors (e.g. using the median for many measurements, rather than the mean), systematic or frequent errors will affect the results. Although manual tracing generally results in fewer errors than automatic segmentation, some species have epidermal cells that are difficult or impossible to observe, regardless of the method of preparation (e.g. because of trichomes, papillae or other cuticular features that obscure the cell walls). Extreme care should be taken if including such species because of the potential to introduce systematic errors in segmentation; for example, if a species has multiple papillae per pavement cell, erroneous tracing of the papillae can result in underestimating cell size.

Automated tracing and annotation of cells to reduce processing time and errors is an exercise in semantic segmentation (see Marmanis *et al*., 2016), and it may be possible to train a machine learning algorithm to perform this step. However, as noted by Vőfély *et al*. (2019), accurate segmentation of images is nontrivial and poses a significant obstacle to high‐throughput studies (though see recent work on stomatal detection by Fetter *et al*., 2019; Aono *et al*., 2021; Xie *et al*., 2021; Li *et al*., 2022). Another obstacle to automation is the multitude of types of epidermal preparation and imaging methods resulting in many different types of input image (see Fig. 1), so we have decoupled epidermalmorph from the challenge of automated segmentation, making it compatible with both algorithmic and manual approaches.

We found that the automated segmentation method implemented in PaCeQuant (Möller *et al*., 2017) performed poorly on our image set (Fig. S8). However, we found that complete segmentation of an image using our hybrid approach took between 10 min and 2 h, and this can be substantially reduced by (1) maximising the quality of the image (avoiding areas where the cuticle is folded/creased or where residual mesophyll is visible beneath the epidermis) and (2) using only the optimal minimum number of cells as described in ‘Sampling effort’ in the Description section. These times to complete segmentation are similar to the time it takes to prepare and capture a high‐quality image of a sample using a light microscope, and much less in some cases, so we do not feel that manual annotation is prohibitively slow. So, while automatic segmentation remains a promising avenue for epidermal image processing (e.g. Berg *et al*., 2019; Aigouy *et al*., 2020) and our dataset of fully annotated images is a valuable source of training data for future endeavours in this area, we suggest that all images should be manually checked and corrected before trait extraction.

Not all traits will be applicable to (or can be reliably measured from) all images or species. This is obvious when cell types are absent in some species in the study group (e.g. no subsidiary cells), but can also be more subtle. For example, stomatal symmetry is not a relevant measurement for species with encircling subsidiary cells (e.g. Fig. 1c,d). In extreme cases of undulation, lobes may occur entirely outside the perimeter of the simplified cell (so undulation frequency may be underestimated) – in these cases, complexity will provide a better measure of undulation. We have included the arguments ‘specific.inclusions’ and ‘specific.exclusions’ in the package to allow measurements to be customised accordingly – see the vignette ‘Measuring different epidermal morphologies with epidermalmorph’ for worked examples. This functionality means that it may also be possible to use epidermalmorph to analyse cells from other tissues (e.g. seed coat and petals), though this was not tested.

### Future development

We envisage that the epidermalmorph package will remain in active development and welcome suggestions for future trait inclusions, potentially including higher‐order spatial measurements (e.g. the Space algorithm; Zeng *et al*., 2020). These suggestions (as well as any user issues) can be lodged under the ‘Issues’ tab on the GitHub page. Although we have not included integrated parallel processing in epidermalmorph, the workflow in the package vignette includes an example of how this might be utilised to increase performance at various steps.

We were unable to design an algorithmic approach to identifying and annotating hypoplastic (nonfunctional) stomata; this presents an opportunity for further development as the presence of hypoplastic stomata is a distinctive feature of some taxa (e.g. *Acmopyle*; Hill & Carpenter, 1991) and quantification of nonfunctional stomata has not, to the best of our knowledge, been studied.

The leaf epidermis contains a wealth of information that is affected by both taxonomic identity and the growing environment. It can be studied in both living and fossil plants to provide insights into plant ecology, physiology and evolution, as well as being useful for palaeoclimatic estimation, but interpretations of epidermal traits remain clouded by convoluted relationships between form, function and ecology. We hope that big‐data approaches to epidermal traits will reveal evolutionary and physiological signals in the epidermis, and here, we provide a method to underpin such studies. Our novel approach to separating gross cell shape from undulation provides more robust measures of undulation that are not affected by cell elongation or nonconvexity. This R package provides a fast and thorough method of quantifying epidermal traits that, with some considerations, can be applied both universally and to targeted groups of plants.

## Author contributions

MJMB and GJJ designed the research and led the writing of the manuscript, MJMB led the development of the R package.

## Supporting information


**Fig. S1** Graphical description of cell simulation algorithm.
**Fig. S2** Measured values of undulation index (UI; see Table 1) on simulated cells.
**Fig. S3** Measured values of solidity (see Table 1) on simulated cells.
**Fig. S4** Measured values of complexity (see Fig. 2) on simulated cells.
**Fig. S5** Measured values of undulation amplitude (see Fig. 2) on simulated cells.
**Fig. S6** Measured values of undulation frequency (see Fig. 2) on simulated cells.
**Fig. S7** Fully labelled version of main text (Fig. 7).
**Fig. S8** Automatic cell segmentation of a high‐quality image of *Podocarpus coriaceus*.
**Methods S1** Image preparation for trait reliability.
**Table S1** Reference list of all traits.
**Table S2** Plants sampled for trait reliability analyses.
Please note: Wiley is not responsible for the content or functionality of any Supporting Information supplied by the authors. Any queries (other than missing material) should be directed to the *New Phytologist* Central Office.

## Data Availability

The R package (including installation instructions) is on GitHub (https://github.com/matildabrown/epidermalmorph) with accompanying documentation and tutorials (https://matildabrown.github.io/epidermalmorph/). Example data are available in the R package as the dataset ‘podocarps’. Figs 7 and 8 are examples of the output produced by this package; the expanded dataset used to generate these figures forms part of a forthcoming study (expected publication in early 2023) and will be made available with this future paper. Please contact the corresponding author for any queries.

## References

[nph18519-bib-0001] Aigouy B , Cortes C , Liu S , Prud'Homme B . 2020. EPySeg: a coding‐free solution for automated segmentation of epithelia using deep learning. Development 147: dev194589. 33268451 10.1242/dev.194589 PMC7774881

[nph18519-bib-0002] Andriankaja M , Dhondt S , De Bodt S , Vanhaeren H , Coppens F , De Milde L , Mühlenbock P , Skirycz A , Gonzalez N , Beemster GT . 2012. Exit from proliferation during leaf development in *Arabidopsis thaliana*: a not‐so‐gradual process. Developmental Cell 22: 64–78. 22227310 10.1016/j.devcel.2011.11.011

[nph18519-bib-0003] Andruchow‐Colombo A , Escapa IH , Carpenter RJ , Hill RS , Iglesias A , Abarzua AM , Wilf P . 2019. Oldest record of the scale‐leaved clade of Podocarpaceae, early Paleocene of Patagonia, Argentina. Alcheringa 43: 127–145.

[nph18519-bib-0004] Aono AH , Nagai JS , Dickel GDS , Marinho RC , de Oliveira PE , Papa JP , Faria FA . 2021. A stomata classification and detection system in microscope images of maize cultivars. PLoS ONE 16: e0258679. 34695146 10.1371/journal.pone.0258679 PMC8544852

[nph18519-bib-0005] Bai Y , Falk S , Schnittger A , Jakoby MJ , Hülskamp M . 2010. Tissue layer specific regulation of leaf length and width in *Arabidopsis* as revealed by the cell autonomous action of ANGUSTIFOLIA. The Plant Journal 61: 191–199. 19843316 10.1111/j.1365-313X.2009.04050.x

[nph18519-bib-0006] Berg S , Kutra D , Kroeger T , Straehle CN , Kausler BX , Haubold C , Schiegg M , Ales J , Beier T , Rudy M . 2019. Ilastik: interactive machine learning for (bio) image analysis. Nature Methods 16: 1226–1232. 31570887 10.1038/s41592-019-0582-9

[nph18519-bib-0007] Bidhendi AJ , Altartouri B , Gosselin FP , Geitmann A . 2019. Mechanical stress initiates and sustains the morphogenesis of wavy leaf epidermal cells. Cell Reports 28: 1237–1250. 31365867 10.1016/j.celrep.2019.07.006

[nph18519-bib-0008] Biffin E , Conran J , Lowe AJ . 2011. Podocarp evolution: a molecular phylogenetic perspective. In: Ecology of the Podocarpaceae in tropical forests, vol. 95. Washington, DC, USA: Smithsonian Contributions to Botany, 1–20.

[nph18519-bib-0009] Blomenkemper P , Bäumer R , Backer M , Abu Hamad A , Wang J , Kerp H , Bomfleur B . 2021. Bennettitalean leaves from the Permian of Equatorial Pangea – the early radiation of an iconic Mesozoic gymnosperm group. Frontiers in Earth Science 9: 162.

[nph18519-bib-0010] Bruschi P , Vendramin GG , Bussotti F , Grossoni P . 2000. Morphological and molecular differentiation between *Quercus petraea* (Matt.) Liebl. and *Quercus pubescens* Willd. (Fagaceae) in northern and central Italy. Annals of Botany 85: 325–333.

[nph18519-bib-0011] Bush RT , Wallace J , Currano ED , Jacobs BF , McInerney FA , Dunn RE , Tabor NJ . 2017. Cell anatomy and leaf δ^13^C as proxies for shading and canopy structure in a Miocene forest from Ethiopia. Palaeogeography, Palaeoclimatology, Palaeoecology 485: 593–604.

[nph18519-bib-0012] Carins Murphy MR , Jordan GJ , Brodribb TJ . 2016. Cell expansion not cell differentiation predominantly co‐ordinates veins and stomata within and among herbs and woody angiosperms grown under sun and shade. Annals of Botany 118: 1127–1138. 27578763 10.1093/aob/mcw167 PMC5963197

[nph18519-bib-0013] Clugston J , Jeffree C , Ahrends A , Mill R . 2017. Do environmental factors affect the taxonomic reliability of leaf cuticular micromorphological characters? A case study in Podocarpaceae. Edinburgh Journal of Botany 74: 299–343.

[nph18519-bib-0014] Deng M , Jiang X‐L , Song Y‐G , Coombes A , Yang X‐R , Xiong Y‐S , Li Q‐S . 2017. Leaf epidermal features of *Quercus* group *Ilex* (Fagaceae) and their application to species identification. Review of Palaeobotany and Palynology 237: 10–36.

[nph18519-bib-0015] Dilcher DL . 1974. Approaches to the identification of angiosperm leaf remains. The Botanical Review 40: 1–157.

[nph18519-bib-0016] Dunn RE , Le TYT , Stromberg CAE . 2015a. Light environment and epidermal cell morphology in grasses. International Journal of Plant Sciences 176: 832–847.

[nph18519-bib-0017] Dunn RE , Stromberg CA , Madden RH , Kohn MJ , Carlini AA . 2015b. Linked canopy, climate, and faunal change in the Cenozoic of Patagonia. Science 347: 258–261. 25593182 10.1126/science.1260947

[nph18519-bib-0018] Farjon A . 2010. A handbook of the world's conifers. Leiden, the Netherlands/Boston, MA, USA: Brill.

[nph18519-bib-0019] Fetter KC , Eberhardt S , Barclay RS , Wing S , Keller SR . 2019. StomataCounter: a neural network for automatic stomata identification and counting. New Phytologist 223: 1671–1681. 31059134 10.1111/nph.15892

[nph18519-bib-0020] Fitzgibbon A , Pilu M , Fisher RB . 1999. Direct least square fitting of ellipses. IEEE Transactions on Pattern Analysis and Machine Intelligence 21: 476–480.

[nph18519-bib-0021] Galletti R , Ingram GC . 2015. Communication is key: reducing DEK1 activity reveals a link between cell‐cell contacts and epidermal cell differentiation status. Communicative & Integrative Biology 8: e1059979. 27064205 10.1080/19420889.2015.1059979 PMC4802766

[nph18519-bib-0022] Gama J . 2015. * conicfit: algorithms for fitting circles*, *ellipses and conics based on the work by Prof. Nikolai Chernov* . R package v.1.0.4. [WWW document] URL https://cran.r‐project.org/web/packages/conicfit/

[nph18519-bib-0090] Hamant O , Heisler MG , Jönsson H , Krupinski P , Uyttewaal M , Bokov P , Corson F , Sahlin P , Boudaoud A , Meyerowitz EM *et al*. 2008. Developmental patterning by mechanical signals in *Arabidopsis* . Science 322: 1650–1655. 19074340 10.1126/science.1165594

[nph18519-bib-0023] Hill R . 1991. Tertiary *Nothofagus* (Fagaceae) macrofossils from Tasmania and Antartica and their bearing on the evolution of the genus. Botanical Journal of the Linnean Society 105: 73–112.

[nph18519-bib-0024] Hill R , Carpenter R . 1991. Evolution of *Acmopyle* and *Dacrycarpus* (Podocarpaceae) foliage as inferred from macrofossils in south‐eastern Australia. Australian Systematic Botany 4: 449–479.

[nph18519-bib-0025] Hill RS , Brodribb TJ . 1999. Turner review no. 2 – Southern conifers in time and space. Australian Journal of Botany 47: 639–696.

[nph18519-bib-0026] Hill RS , Pole MS . 1992. Leaf and shoot morphology of extant *Afrocarpus*, *Nageia* and *Retrophyllum* (Podocarpaceae) species, and species with similar leaf arrangement, from Tertiary sediments in Australasia. Australian Systematic Botany 5: 337–358.

[nph18519-bib-0027] Jacques E , Verbelen J‐P , Vissenberg K . 2014. Review on shape formation in epidermal pavement cells of the *Arabidopsis* leaf. Functional Plant Biology 41: 914–921. 32481044 10.1071/FP13338

[nph18519-bib-0029] Jordan GJ . 2011. A critical framework for the assessment of biological palaeoproxies: predicting past climate and levels of atmospheric CO_2_ from fossil leaves. New Phytologist 192: 29–44. 21770947 10.1111/j.1469-8137.2011.03829.x

[nph18519-bib-0030] Jordan GJ , Hill RS . 1999. The phylogenetic affinities of *Nothofagus* (Nothofagaceae) leaf fossils based on combined molecular and morphological data. International Journal of Plant Sciences 160: 1177–1188. 10568786 10.1086/314207

[nph18519-bib-0032] Kubínová L . 1994. Recent stereological methods for measuring leaf anatomical characteristics: estimation of the number and sizes of stomata and mesophyll cells. Journal of Experimental Botany 45: 119–127.

[nph18519-bib-0033] Kürschner WM . 1997. The anatomical diversity of recent and fossil leaves of the durmast oak (*Quercus petraea* Lieblein/*Q. pseudocastanea* Goeppert) – implications for their use as biosensors of palaeoatmospheric CO_2_ levels. Review of Palaeobotany and Palynology 96: 1–30.

[nph18519-bib-0034] Leslie AB , Beaulieu J , Holman G , Campbell CS , Mei W , Raubeson LR , Mathews S . 2018. An overview of extant conifer evolution from the perspective of the fossil record. American Journal of Botany 105: 1531–1544. 30157290 10.1002/ajb2.1143

[nph18519-bib-0035] Li S , Li L , Fan W , Ma S , Zhang C , Kim JC , Wang K , Russinova E , Zhu Y , Zhou Y . 2022. LeafNet: a tool for segmenting and quantifying stomata and pavement cells. Plant Cell 34: 1171–1188. 35080620 10.1093/plcell/koac021 PMC8972303

[nph18519-bib-0036] Marmanis D , Wegner JD , Galliani S , Schindler K , Datcu M , Stilla U . 2016. Semantic segmentation of aerial images with an ensemble of CNSS. ISPRS Annals of the Photogrammetry, Remote Sensing and Spatial Information Sciences 3: 473–480.

[nph18519-bib-0037] McElwain JC , Montañez I , White JD , Wilson JP , Yiotis C . 2016. Was atmospheric CO_2_ capped at 1000 ppm over the past 300 million years? Palaeogeography, Palaeoclimatology, Palaeoecology 441: 653–658.

[nph18519-bib-0038] McLellan T , Endler JA . 1998. The relative success of some methods for measuring and describing the shape of complex objects. Systematic Biology 47: 264–281.

[nph18519-bib-0039] Metcalfe C , Chalk L . 1979. Anatomy of the dicotyledons. Vol I. Systematic anatomy of leaf and stem, with a brief history of the subject. Oxford, UK: Oxford Science.

[nph18519-bib-0040] Möller B , Poeschl Y , Plötner R , Bürstenbinder K . 2017. PaCeQuant: a tool for high‐throughput quantification of pavement cell shape characteristics. Plant Physiology 175: 998–1017. 28931626 10.1104/pp.17.00961 PMC5664455

[nph18519-bib-0041] Mosbrugger V , Utescher T . 1997. The coexistence approach – a method for quantitative reconstructions of Tertiary terrestrial palaeoclimate data using plant fossils. Palaeogeography, Palaeoclimatology, Palaeoecology 134: 61–86.

[nph18519-bib-0042] Nowak J , Eng RC , Matz T , Waack M , Persson S , Sampathkumar A , Nikoloski Z . 2021. A network‐based framework for shape analysis enables accurate characterization of leaf epidermal cells. Nature Communications 12: 1–13. 10.1038/s41467-020-20730-y PMC7815848 33469016

[nph18519-bib-0043] Osunkoya OO , Boyne R , Scharaschkin T . 2014. Coordination and plasticity in leaf anatomical traits of invasive and native vine species. American Journal of Botany 101: 1423–1436. 25253703 10.3732/ajb.1400125

[nph18519-bib-0044] Page CN . 2019. New and maintained genera in the taxonomic alliance of *Prumnopitys* s. l. (Podocarpaceae), and circumscription of a new genus: *Pectinopitys* . New Zealand Journal of Botany 57: 137–153.

[nph18519-bib-0045] de Reuille PB , Routier‐Kierzkowska AL , Kierzkowski D , Bassel GW , Schüpbach T , Tauriello G , Bajpai N , Strauss S , Weber A , Kiss A *et al*. 2015. MorphoGraphX: a platform for quantifying morphogenesis in 4D. eLife 4: e05864. 25946108 10.7554/eLife.05864 PMC4421794

[nph18519-bib-0046] Royer D . 2001. Stomatal density and stomatal index as indicators of paleoatmospheric CO_2_ concentration. Review of Palaeobotany and Palynology 114: 1–28. 11295163 10.1016/s0034-6667(00)00074-9

[nph18519-bib-0047] Sánchez‐Corrales YE , Hartley M , Van Rooij J , Marée AF , Grieneisen VA . 2018. Morphometrics of complex cell shapes: lobe contribution elliptic Fourier analysis (LOCO‐EFA). Development 145: dev156778. 29444894 10.1242/dev.156778 PMC5897594

[nph18519-bib-0048] Sapala A , Runions A , Smith RS . 2019. Mechanics, geometry and genetics of epidermal cell shape regulation: different pieces of the same puzzle. Current Opinion in Plant Biology 47: 1–8. 30170216 10.1016/j.pbi.2018.07.017

[nph18519-bib-0049] Sharma G . 1972. Environmental modifications of leaf epidermis and morphological features in *Verbena canadensis* . The Southwestern Naturalist 17: 221–228.

[nph18519-bib-0051] Sotiriou P , Giannoutsou E , Panteris E , Galatis B , Apostolakos P . 2018. Local differentiation of cell wall matrix polysaccharides in sinuous pavement cells: its possible involvement in the flexibility of cell shape. Plant Biology 20: 223–237. 29247575 10.1111/plb.12681

[nph18519-bib-0052] Stark Schilling DM , Mill RR . 2011. Cuticle micromorphology of Caribbean and central american species of *Podocarpus* (Podocarpaceae). International Journal of Plant Sciences 172: 601–631.

[nph18519-bib-0053] Steger C . 1998. An unbiased detector of curvilinear structures. IEEE Transactions on Pattern Analysis and Machine Intelligence 20: 113–125.

[nph18519-bib-0054] Strimas‐Mackey ME . 2021. smoothr: smooth and tidy spatial features . R package v.0.2.1. [WWW document] URL https://cran.r‐project.org/web/packages/smoothr/

[nph18519-bib-0055] Thomas PW , Woodward FI , Quick WP . 2004. Systemic irradiance signalling in tobacco. New Phytologist 161: 193–198.

[nph18519-bib-0056] Thorsten Wagner MH , xraynaud . 2017. Ridge detection. *Zenodo*. doi: 10.5281/zenodo.845874.

[nph18519-bib-0058] Vőfély RV , Gallagher J , Pisano GD , Bartlett M , Braybrook SA . 2019. Of puzzles and pavements: a quantitative exploration of leaf epidermal cell shape. New Phytologist 221: 540–552. 30281798 10.1111/nph.15461 PMC6585845

[nph18519-bib-0059] Wells PM , Hill RS . 1989. Leaf morphology of the imbricate‐leaved Podocarpaceae. Australian Systematic Botany 2: 369–386.

[nph18519-bib-0060] Wickham H. 2016. ggplot2: elegant graphics for data analysis. New York, NY, USA: Springer‐Verlag. [WWW document] URL https://ggplot2.tidyverse.org

[nph18519-bib-0061] Wilf P , Zhang S , Chikkerur S , Little SA , Wing SL , Serre T . 2016. Computer vision cracks the leaf code. Proceedings of the National Academy of Sciences, USA 113: 3305–3310. 10.1073/pnas.1524473113 PMC4812720 26951664

[nph18519-bib-0062] Xie J , Fernandes SB , Mayfield‐Jones D , Erice G , Choi M , Lipka EA , Leakey AD . 2021. Optical topometry and machine learning to rapidly phenotype stomatal patterning traits for maize QTL mapping. Plant Physiology 187: 1462–1480. 34618057 10.1093/plphys/kiab299 PMC8566313

[nph18519-bib-0063] Zeng SM , Lo EK , Hazelton BJ , Morales MF , Torii KU . 2020. Effective range of non‐cell autonomous activator and inhibitor peptides specifying plant stomatal patterning. Development 147: dev192237. 32816968 10.1242/dev.192237 PMC7502594

