# Supplementary material for: No cell is an island: characterising the leaf epidermis using epidermalmorph, a new R package
Source: New Phytol. 2022 Nov 15;237(1):354–66. doi: 10.1111/nph.18519 (PMC10098627; doi:10.1111/nph.18519)
Supplement: Supplementary file 1 — Fig. S1 Graphical description of cell simulation algorithm. Fig. S2 Measured values of undulation index (UI; see Table 1) on simulated cells. Fig. S3 Measured values of solidity (see Table 1) on simulated cells. Fig. S4 Measured values of complexity (see Fig. 2) on simulated cells. Fig. S5 Measured values of undulation amplitude (see Fig. 2) on simulated cells. Fig. S6 Measured values of undulation frequency (see Fig. 2) on simulated cells. Fig. S7 Fully labelled version of main text (Fig. 7). Fig. S8 Automatic cell segmentation of a high‐quality image of Podocarpus coriaceus. Methods S1 Image preparation for trait reliability. Table S1 Reference list of all traits. Table S2 Plants sampled for trait reliability analyses. Please note: Wiley is not responsible for the content or functionality of any Supporting Information supplied by the authors. Any queries (other than missing material) should be directed to the New Phytologist Central Office. [file NPH-237-354-s001.pdf]

# Supporting Information for: ‘No cell is an island: characterising the leaf epidermis using EPIDERMALMORPH, a new R package’

Matilda J.M. Brown & Gregory J. Jordan

**Article accepted:** 12 September 2022

---

**Contents**

**Methods S1** – Image preparation for trait reliability.

**Table S1** Reference list of all traits

**Table S2** Plants sampled for trait reliability analyses.

**Figure S1** Graphical description of cell simulation algorithm.

**Figure S2** Measured values of undulation index (UI; see Table 1) on simulated cells.

**Figure S3** Measured values solidity (see Table 1) on simulated cells.

**Figure S4** Measured values of complexity (see Figure 2) on simulated cells.

**Figure S5** Measured values of undulation amplitude (see Figure 2) on simulated cells.

**Figure S6** Measured values of undulation frequency (see Figure 2) on simulated cells.

**Figure S7** Fully labelled version of main text Figure 7

**Figure S8** Automatic cell segmentation of a high-quality image of *Podocarpus coriaceus.*

---

**Methods S1 –** Image preparation for trait reliability

Fully expanded adult leaves were collected from healthy plants grown at the University of Tasmania and the Royal Botanic Gardens Edinburgh (Table S1). These plants were all growing in shaded, frost-free greenhouses. For species with a wide geographic distribution, we sampled leaves from individuals with multiple provenances (where this was possible).

For large leaves, pieces of approximately 1cm$^2$ were cut from either side of the midrib in the middle third of the leaf; for smaller leaves, the base, apex and, if possible, the margins were removed. These samples were soaked in commercial household bleach (50 gL$^{-1}$ sodium hypochlorite and 13 gL$^{-1}$ sodium hydroxide) until the cuticle separated from the mesophyll. Bleach was removed by thoroughly rinsing in water and remaining mesophyll tissue was removed using a fine paintbrush. Sections were stained with 1% crystal violet or safranin solution for 1 minute, then mounted in phenol glycerine jelly. Several fields of view at ×10 magnification (field of view area, 0.56mm$^2$) were photographed from each section using a Nikon Digital Sight DS-L1 camera (Melville, NY, USA) mounted on a Leica DM 1000 microscope (Nussloch, Germany). The best 3-5 images (identified as the clearest, without damage or obvious distortion) for each individual plant were selected for analyses. Image pre-processing and annotation was done in ImageJ. Images were converted to 8-bit grayscale, then Ridge Detection was performed (parameters manually set for each image to give best results) to binarize the image. For high-resolution images, we dilated the resulting image to better see the cell walls (1px wide → 3px wide). We then used the paintbrush tool to correct the tracing, and the flood fill tool to annotate cell types (we used a value of 85 for stomata, 170 for subsidiary cells and 50 for salt glands, but these are arbitrary and any values could be used). Images were then further analysed with EPIDERMALMORPH as described in the main text.

**Table S1.** Complete reference of all traits measured by epidermalmorph. This is available in the package as 'trait_key' and can be used to filter traits by cell type, category, scale dependency and statistic (sd or mean/median).

| trait (alphabetically sorted after number of cells of each type) | celltype | category | sd | scale depe-ndent | description |
|---|---|---|---|---|---|
| image.ID | | basic | 0 | 0 | Identifier for image |
| rotation | | basic | 0 | 0 | Rotation of image, either stomatal north or the user input rotation |
| n.pavement | | number | 0 | 1 | Number of pavement cells |
| n.stomata | stomata | number | 0 | 1 | Number of stomata |
| n.subsidiary | subsidiary | number | 0 | 1 | Number of subsidiary cells |
| n.other | other | number | 0 | 1 | Number of other cells |
| dist.between.stom.rows | stomata | stomatal arrangement | 0 | 1 | Distance between peaks in stomatal density |
| other.area.median | other | other cell | 0 | 1 | Median area of 'other' cells |
| other.density | other | other cell | 0 | 1 | Density (number per area) of 'other' cells |
| other.distNN.mean | other | other cell | 0 | 1 | Mean distance between (groups of) 'other' cells |
| other.grouping | other | other cell | 0 | 0 | Grouping of 'other' cells - 1 if cells occur individually, 2, if in adjacent pairs, etc. |
| other.index | other | other cell | 0 | 0 | Number of other cells (specific type can be named) as proportion of all cells |
| other.spacingNN.mean | other | other cell | 0 | 0 | Mean number of cells between (groups of) 'other' cells |
| pavezone.angle.median | pavezone | pavement | 0 | 0 | Median angle (relative to rotation/stomatal north) of pavezone cells (i.e. >2 cells from stomata and thus outside the zone of shape distortion) |
| pavezone.angle.sd | pavezone | pavement | 1 | 0 | Standard deviation of the angle (relative to rotation/stomatal north) of pavezone cells |
| pavezone.AR.median | pavezone | pavement | 0 | 0 | Median aspect ratio of pavezone cells (i.e. >2 cells from stomata and thus outside the zone of shape distortion) |
| pavezone.AR.sd | pavezone | pavement | 1 | 0 | Standard deviation of the aspect ratio of pavezone cells (i.e. >2 cells from stomata and thus outside the zone of shape distortion) |
| pavezone.area.median | pavezone | pavement | 0 | 1 | Median area of pavezone cells (i.e. >2 cells from stomata and thus outside the zone of shape distortion) |
| pavezone.area.sd | pavezone | pavement | 1 | 1 | Standard deviation of the area of pavezone cells (i.e. >2 cells from stomata and thus outside the zone of shape distortion) |
| pavezone.complexity.median | pavezone | pavement | 0 | 0 | Median complexity (perimeter/simplified perimeter) of pavezone cells (i.e. >2 cells from stomata and thus outside the zone of shape distortion) |
| pavezone.complexity.sd | pavezone | pavement | 1 | 0 | Standard deviation of complexity (perimeter/simplified perimeter) of pavezone cells (i.e. >2 cells from stomata and thus outside the zone of shape distortion) |

| | | | | | |
|---|---|---|---|---|---|
| pavezone.endwallangle.mean | pavezone | pavement | 0 | 0 | Mean angle of the endwalls of pavezone cells (i.e. >2 cells from stomata and thus outside the zone of shape distortion) |
| pavezone.endwalldiff.mean | pavezone | pavement | 0 | 0 | Mean difference in angle of the endwalls of pavezone cells (i.e. >2 cells from stomata and thus outside the zone of shape distortion) |
| pavezone.njunctionpts.mean | pavezone | pavement | 0 | 0 | Mean number of junction points (i.e. number of neighbours) of pavezone cells (i.e. >2 cells from stomata and thus outside the zone of shape distortion) |
| pavezone.njunctionpts.sd | pavezone | pavement | 1 | 0 | Standard deviation of the number of junction points (i.e. number of neighbours) of pavezone cells (i.e. >2 cells from stomata and thus outside the zone of shape distortion) |
| pavezone.undulation.amp.median | pavezone | pavement | 0 | 1 | Median maximum undulation amplitude of pavezone cells (i.e. >2 cells from stomata and thus outside the zone of shape distortion) |
| pavezone.undulation.amp.sd | pavezone | pavement | 1 | 1 | Standard deviation of the maximum undulation amplitude of pavezone cells (i.e. >2 cells from stomata and thus outside the zone of shape distortion) |
| pavezone.undulation.freq.mean | pavezone | pavement | 0 | 1 | Mean undulation frequency of pavezone cells (i.e. >2 cells from stomata and thus outside the zone of shape distortion) |
| pavezone.undulation.freq.sd | pavezone | pavement | 1 | 1 | Standard deviation of the undulation frequency of pavezone cells (i.e. >2 cells from stomata and thus outside the zone of shape distortion) |
| polar.AR.median | polar | pavement | 0 | 0 | Median aspect ratio of polar cells (i.e. adjacent to stomata but not labelled as subsidiary cells) |
| polar.area.median | polar | pavement | 0 | 1 | Median area of polar cells (i.e. adjacent to stomata but not labelled as subsidiary cells) |
| polar.area.sd | polar | pavement | 1 | 1 | Standard deviation of the area of polar cells (i.e. adjacent to stomata but not labelled as subsidiary cells) |
| polar.complexity.median | polar | pavement | 0 | 0 | Median complexity (perimeter/simplified perimeter) of polar cells (i.e. adjacent to stomata but not labelled as subsidiary cells) |
| polar.undulation.amp.median | polar | pavement | 0 | 1 | Median maximum undulation amplitude of polar cells (i.e. adjacent to stomata but not labelled as subsidiary cells) |
| polar.undulation.freq.mean | polar | pavement | 0 | 1 | Mean undulation frequency of polar cells (i.e. adjacent to stomata but not labelled as subsidiary cells) |
| row.consistency | stomata | stomatal arrangement | 0 | 1 | Standard deviation of the number of stomata in each row (rows assigned using density perpendicular to the rotation/stomatal north) |
| row.wiggliness | stomata | stomatal arrangement | 0 | 0 | Proportion of variance explained by PC2 in the distribution of stomatal centroids in each row (rows assigned using density perpendicular to the rotation/stomatal north). 0 = perfect alignment; 0.5 = rows as wide as they are long (i.e., no alignment) |
| stom.angle.sd | stomata | stomatal arrangement | 0 | 0 | Standard deviation of the angles of stomata |
| stom.AR.mean | stomata | stomatal shape | 0 | 0 | Mean stomatal aspect ratio (length/width) |

| | | | | | |
|---|---|---|---|---|---|
| stom.AR.sd | stomata | stomatal shape | 1 | 0 | Standard deviation of stomatal aspect ratios (length/width) |
| stom.butterfly.mean | stomata | stomatal shape | 0 | 0 | Ratio of guard cell length to subsidiary cell length - do lateral subsidiary cells extend beyond the guard cells, making the stomatal complex look like a butterfly? |
| stom.dist2NN.mean | stomata | stomatal arrangement | 0 | 1 | Mean distance (in pixels or other units) between each stomata and its second nearest neighbour |
| stom.dist2NN.sd | stomata | stomatal arrangement | 1 | 1 | Standard deviation of the distances (in pixels or other units) between each stomata and its second nearest neighbour |
| stom.distNN.mean | stomata | stomatal arrangement | 0 | 1 | Mean distance (in pixels or other units) between each stomata and its nearest neighbour |
| stom.distNN.sd | stomata | stomatal arrangement | 1 | 1 | Standard deviation of the distances (in pixels or other units) between each stomata and its nearest neighbour |
| stom.gclength.mean | stomata | stomatal shape | 0 | 1 | Mean guard cell length (length of longest axis of stomate) |
| stom.gclength.sd | stomata | stomatal shape | 1 | 1 | Standard deviation of guard cell lengths (length of longest axis of stomate) |
| stom.nsubcells.mean | stomata | stomatal shape | 0 | 0 | Mean number of subsidiary cells per stomate |
| stom.spacingNN.mean | stomata | stomatal arrangement | 0 | 0 | Mean number of cells between each stomata and its nearest neighbour |
| stom.spacingNN.sd | stomata | stomatal arrangement | 1 | 0 | Standard deviation of the numbers of cells between each stomata and its nearest neighbour |
| stom.subsarea.mean | stomata | stomatal shape | 0 | 1 | Mean subsidiary cell area per stomate |
| stom.symmetry.mean | stomata | stomatal shape | 0 | 0 | Ratio of area of larger subsidiary cell:smaller subsidiary cell. |
| stomatal.density | stomata | stomata basic | 0 | 1 | Stomatal density (stomata per area; in pixels or other unit; depending on image scale) |
| stomatal.index | stomata | stomata basic | 0 | 0 | Number of stomata as a proportion of total number of cells |
| stomzone.AR.median | stomzone | pavement | 0 | 0 | Median aspect ratio of stomzone cells (i.e. 2 cells from stomata and thus inside the zone of shape distortion) |
| stomzone.area.median | stomzone | pavement | 0 | 1 | Median area of stomzone cells (i.e. 2 cells from stomata and thus inside the zone of shape distortion) |
| stomzone.area.sd | stomzone | pavement | 1 | 1 | Standard deviation of the area of stomzone cells (i.e. 2 cells from stomata and thus inside the zone of shape distortion) |
| stomzone.complexity.median | stomzone | pavement | 0 | 0 | Median complexity (perimeter/simplified perimeter) of stomzone cells (i.e. 2 cells from stomata and thus inside the zone of shape distortion) |
| stomzone.undulation.amp.median | stomzone | pavement | 0 | 1 | Median maximum undulation amplitude of stomzone cells (i.e. 2 cells from stomata and thus inside the zone of shape distortion) |
| stomzone.undulation.freq.mean | stomzone | pavement | 0 | 1 | Mean undulation frequency of stomzone cells (i.e. 2 cells from stomata and thus inside the zone of shape distortion) |

**Table S2** Plants sampled for trait reliability analyses. Plants were sampled from the Royal Botanical Gardens, Edinburgh (RBGE) or the University of Tasmania, Hobart (UTAS).

| PLANT ID | SPECIES | COLLECTED FROM | ACCESSION # |
|---|---|---|---|
| **ACMOPYLE_PANCHERI1** | *Acmopyle pancheri* | RBGE | 19842681 |
| **AFROCARPUS_FALCATUS1** | *Afrocarpus falcatus* | RBGE | 20001613 |
| **AFROCARPUS_GRACILIOR1** | *Afrocarpus gracilior* | RBGE | 19820027 |
| **AFROCARPUS_MANNII1** | *Afrocarpus mannii* | RBGE | 19960587 |
| **FALCATIFOLIUM_TAXOIDES1** | *Falcatifolium taxoides* | UTAS | - |
| **FALCATIFOLIUM_TAXOIDES2** | *Falcatifolium taxoides* | UTAS | - |
| **PECTINOPITYS_LADEI1** | *Pectinopitys ladei* | UTAS | - |
| **PHYLLOCLADUS_ASPLENIIFOLIUS1** | *Phyllocladus aspleniifolius* | UTAS | - |
| **PHYLLOCLADUS_TRICHOMANOIDES1** | *Phyllocladus trichomanoides* | UTAS | - |
| **PODOCARPUS_BRASSII1** | *Podocarpus brassii* | UTAS | - |
| **PODOCARPUS_CORIACEUS1** | *Podocarpus coriaceus* | RBGE | 20030490 |
| **PODOCARPUS_COSTALIS1** | *Podocarpus costalis* | RBGE | 19763956 |
| **PODOCARPUS_DISPERMUS1** | *Podocarpus dispermus* | UTAS | - |
| **PODOCARPUS_DISPERMUS2** | *Podocarpus dispermus* | RBGE | 20110038 |
| **PODOCARPUS_FORRESTII1** | *Podocarpus forrestii* | RBGE | 19915024 |
| **PODOCARPUS_LUCIENII1** | *Podocarpus lucienii* | RBGE | 20010205 |
| **PODOCARPUS_MATUDAE1** | *Podocarpus matudae* | RBGE | 19972324 |
| **PODOCARPUS_NAKAII1** | *Podocarpus nakaii* | RBGE | 19763844 |
| **PODOCARPUS_NERIIFOLIUS1** | *Podocarpus neriifolius* | UTAS | - |
| **PODOCARPUS_NERIIFOLIUS2** | *Podocarpus neriifolius* | RBGE | 19681468 |
| **PODOCARPUS_PILGERI1** | *Podocarpus pilgeri* | RBGE | 20022521 |
| **PODOCARPUS_SELLOWII1** | *Podocarpus sellowii* | RBGE | 20071743 |
| **PODOCARPUS_TRINITENSIS1** | *Podocarpus trinitensis* | RBGE | 20030491 |
| **RETROPHYLLUM_ROSPIGLIOSSII1** | *Retrophyllum rospigliossii* | RBGE | 19951953 |
| **RETROPHYLLUM_ROSPIGLIOSSII2** | *Retrophyllum rospigliossii* | UTAS | - |
| **SUNDACARPUS_AMARUS1** | *Sundacarpus amarus* | RBGE | 20030752 |

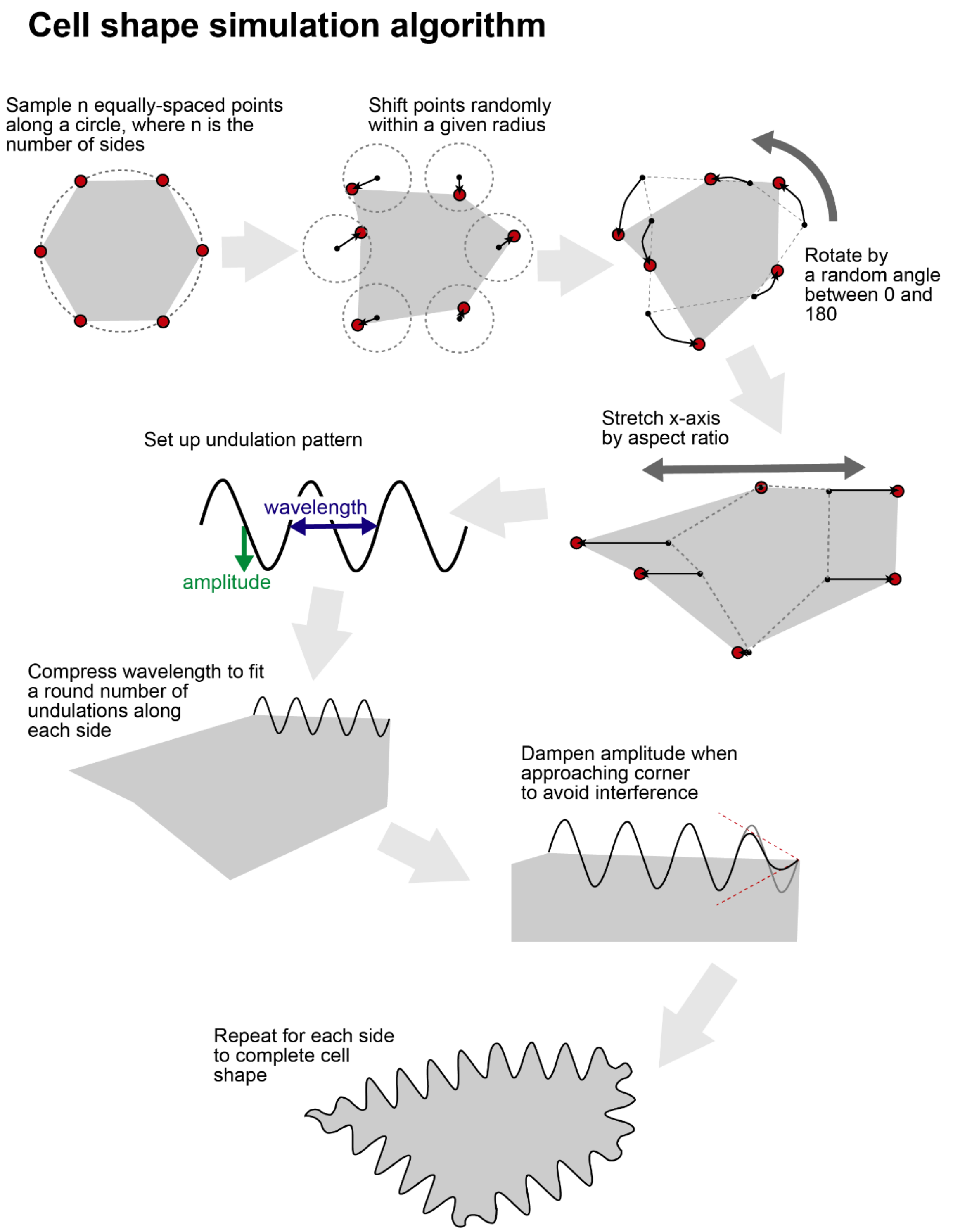


**Figure S1.** Simulated cell generation. We generated cells with 3,4,5 and 6 sides, with aspect ratios of 1,2 and 5. These formed the 'straight-walled' cells. Each of these cells was then undulated with a frequency of 1, 2 and 3, and an amplitude of 0.2, 1 and 2, although for some shapes the maximum amplitude was not reached because of dampening effects.

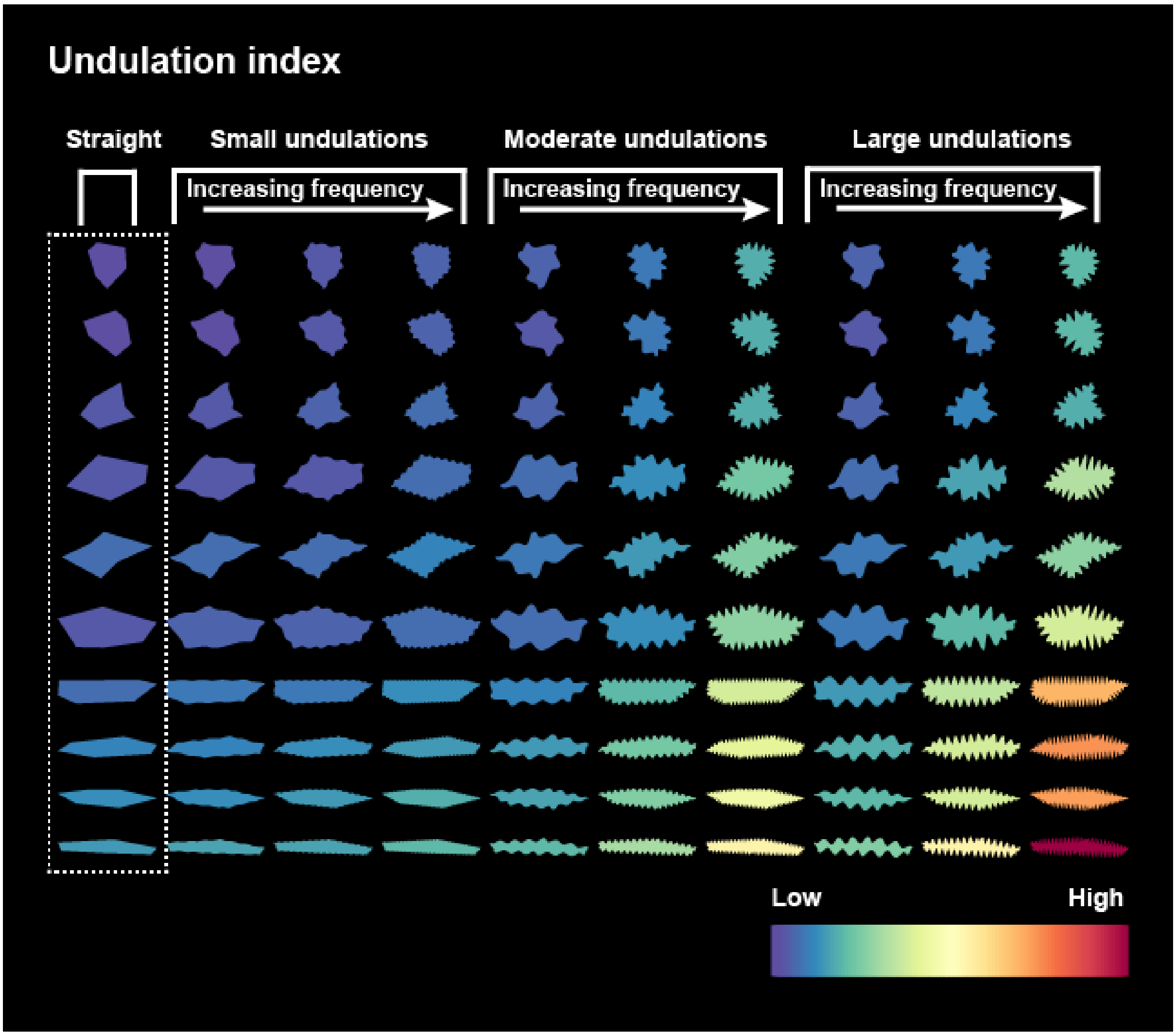


**Figure S2.** Measured values of undulation index (UI; see Table 1) on simulated cells. On straight-walled cells (highlighted with white dotted line), UI increases with aspect ratio.

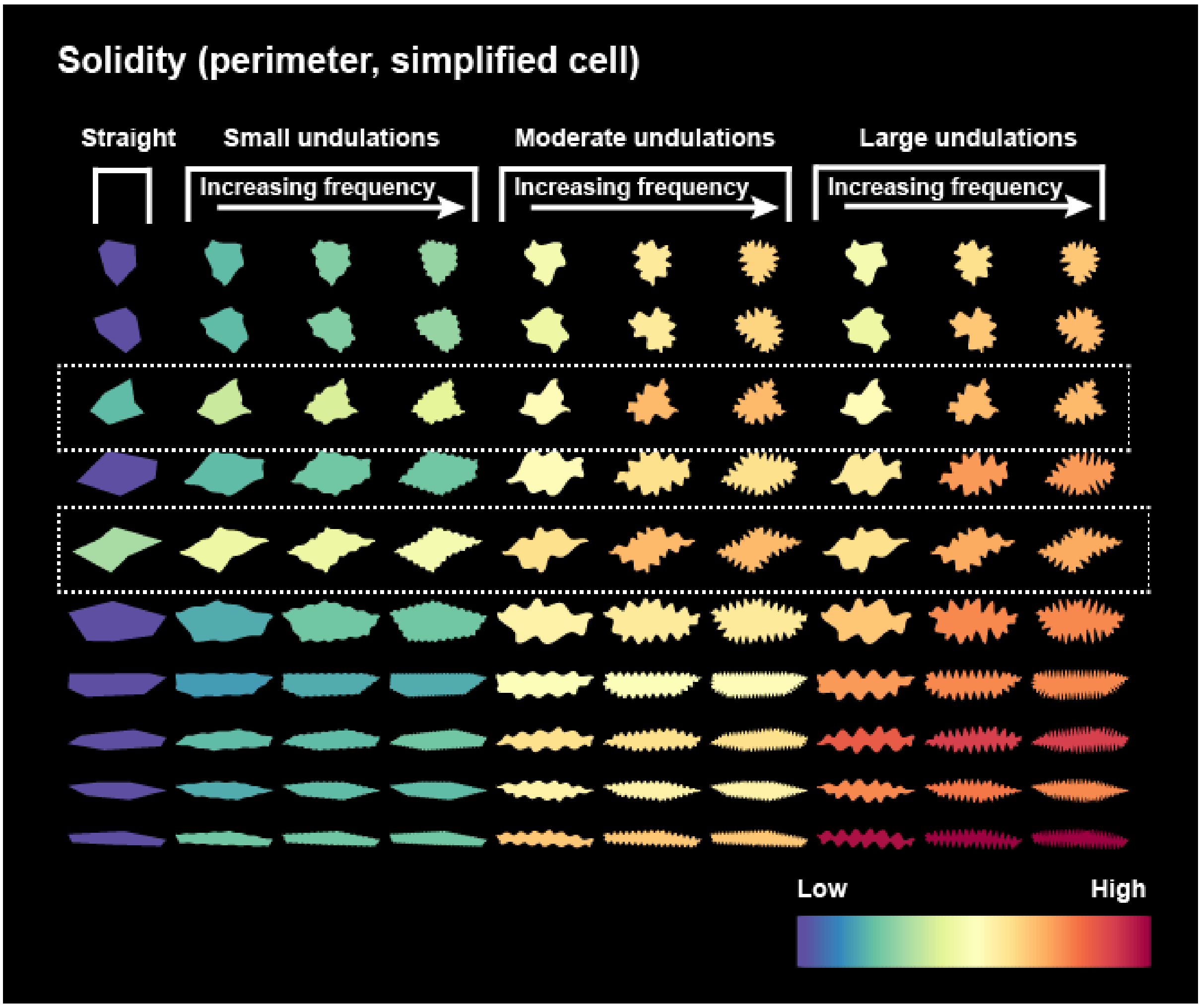


**Figure S3.** Measured values of solidity (see Table 1) on simulated cells. Solidity values are inflated for non-convex cells (highlighted with white dotted lines).

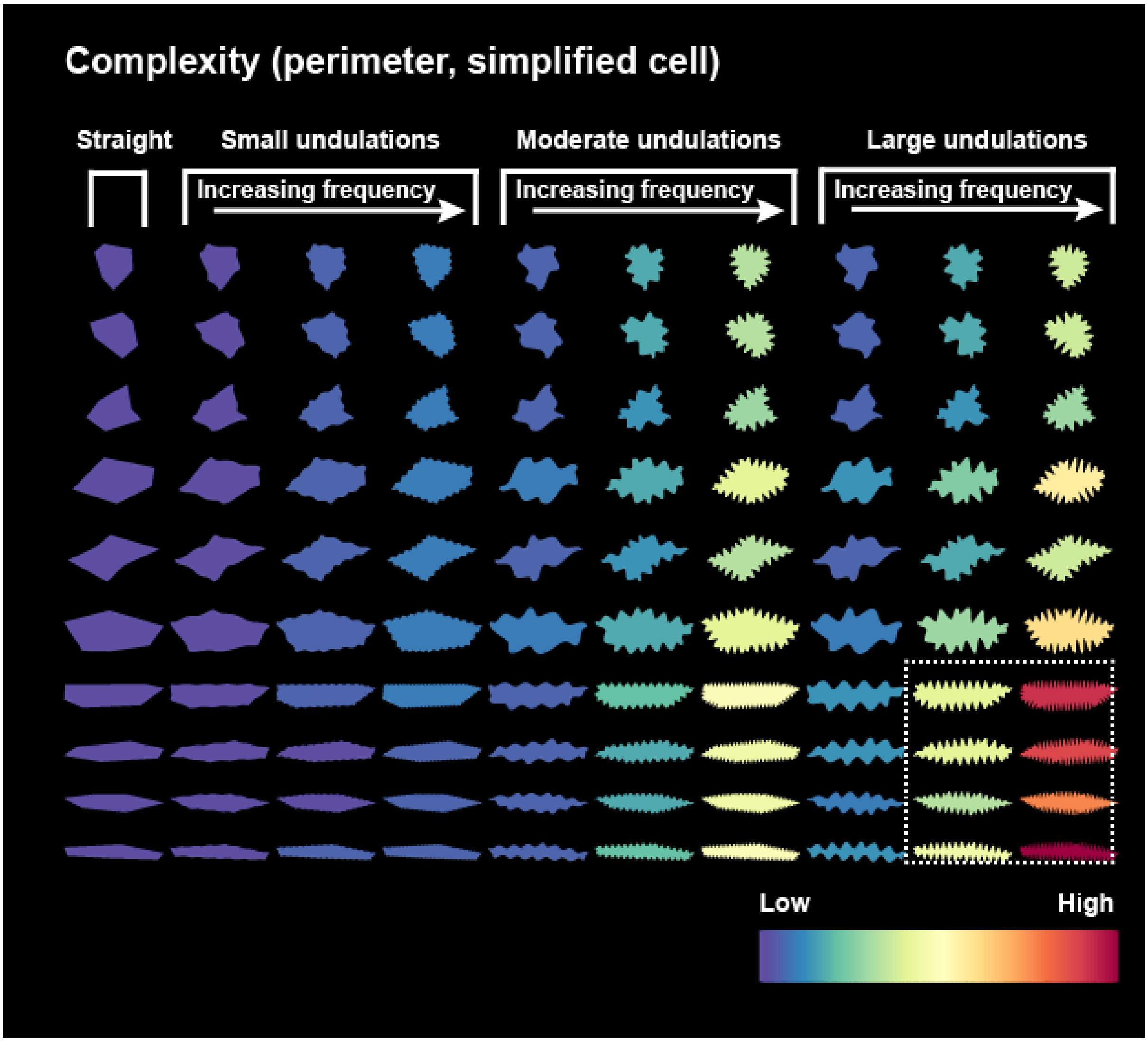


**Figure S4**. Measured values of complexity, a new metric presented here (see Figure 2), on simulated cells. Increases with undulation size and with undulation frequency. Note that the highlighted cells (enclosed by dotted line) have exceptionally high values – this is due to the non-linear relationship between undulated and simple perimeter.

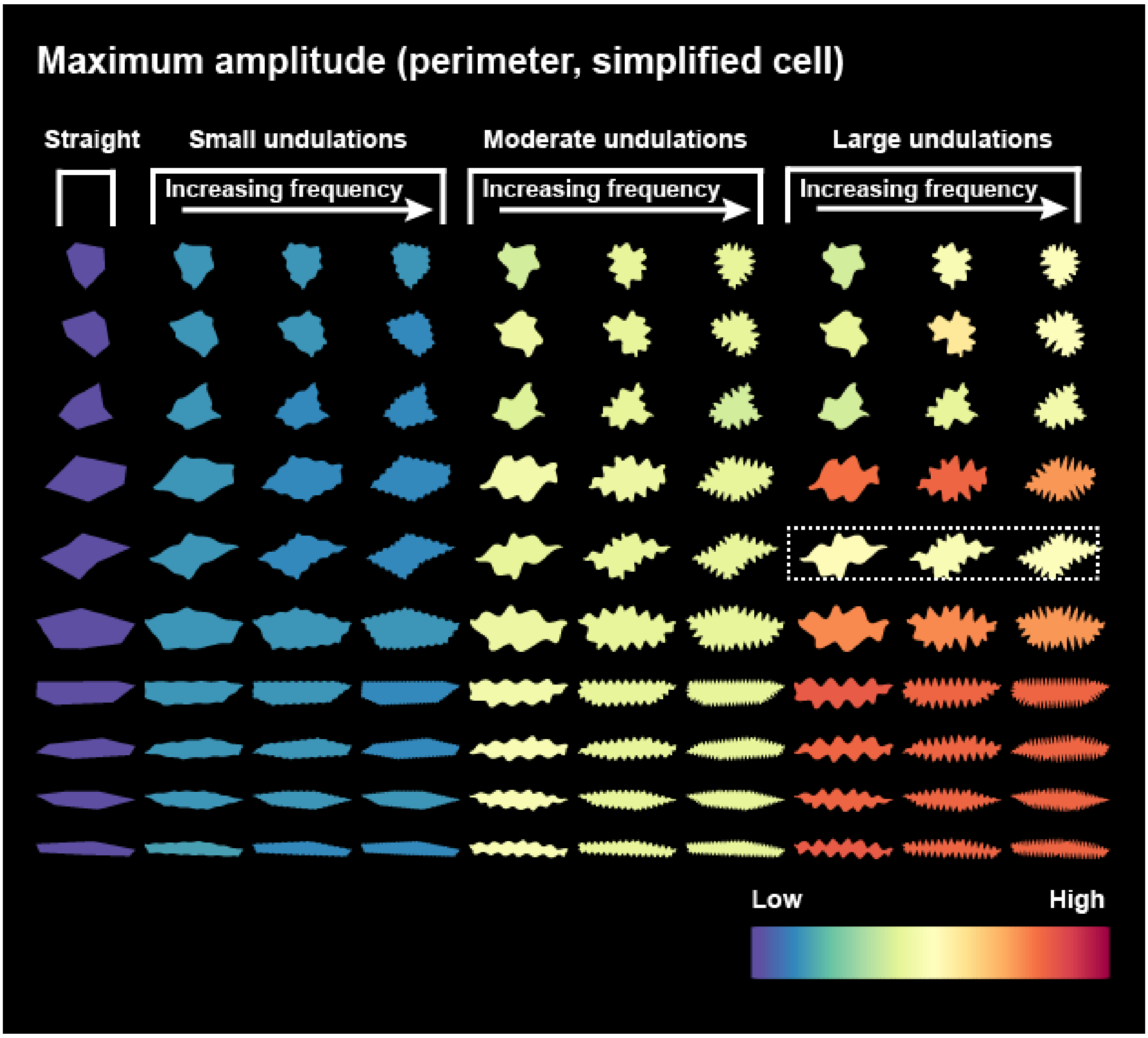


**Figure S5.** Measured values of maximum amplitude, a new metric presented here (see Fig. 2), on simulated cells. Increases with undulation size. Note that for some cells (e.g. those highlighted by the dotted line), the shape of the cell precludes the maximum amplitude from being reached – this is a quirk of the cell generation, not of the metric.

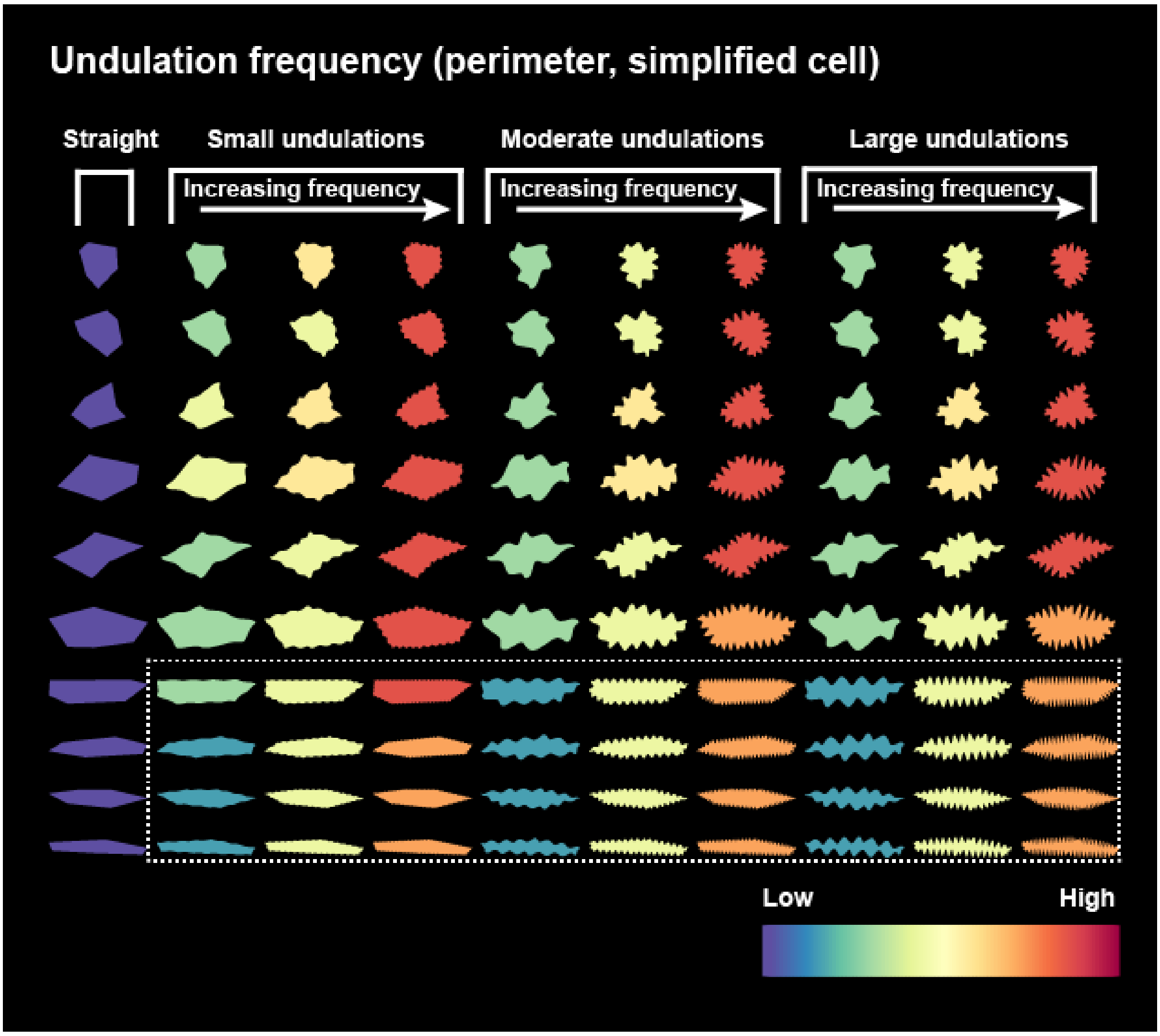


**Figure S6**. Measured values of undulation frequency, a new metric presented here (see Fig. 2), on simulated cells. Increases with undulation frequency. Note that for some cells (e.g. those highlighted by the dotted line), the frequency is lower than expected – this is a quirk of the cell generation (specifically the rounding of the wavelength to fit an integer number of undulations along a side), not of the metric.

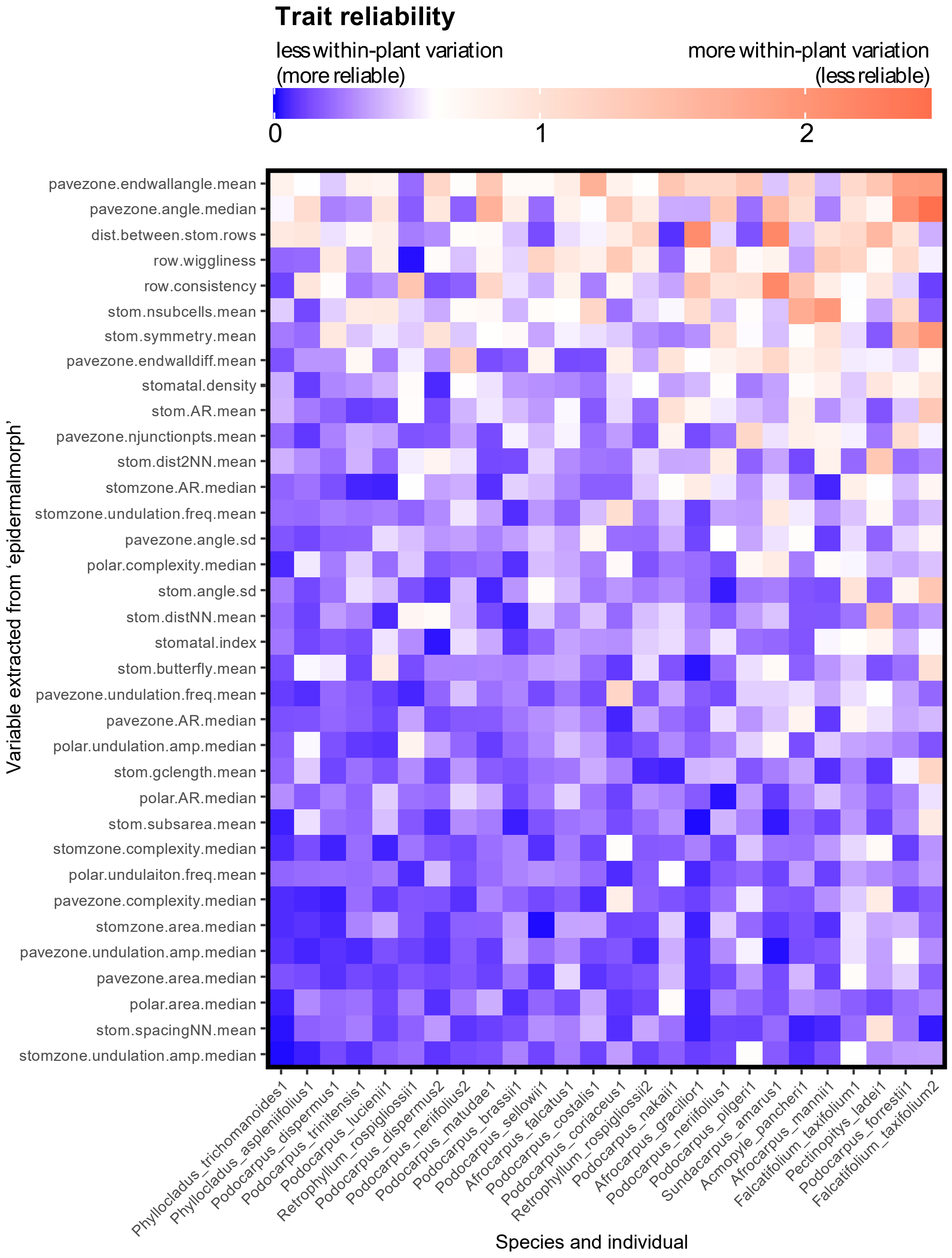


**Figure S7.** Fully labelled version of Figure 7, with traits shown as they occur in the output of 'extract_epidermal_traits'.

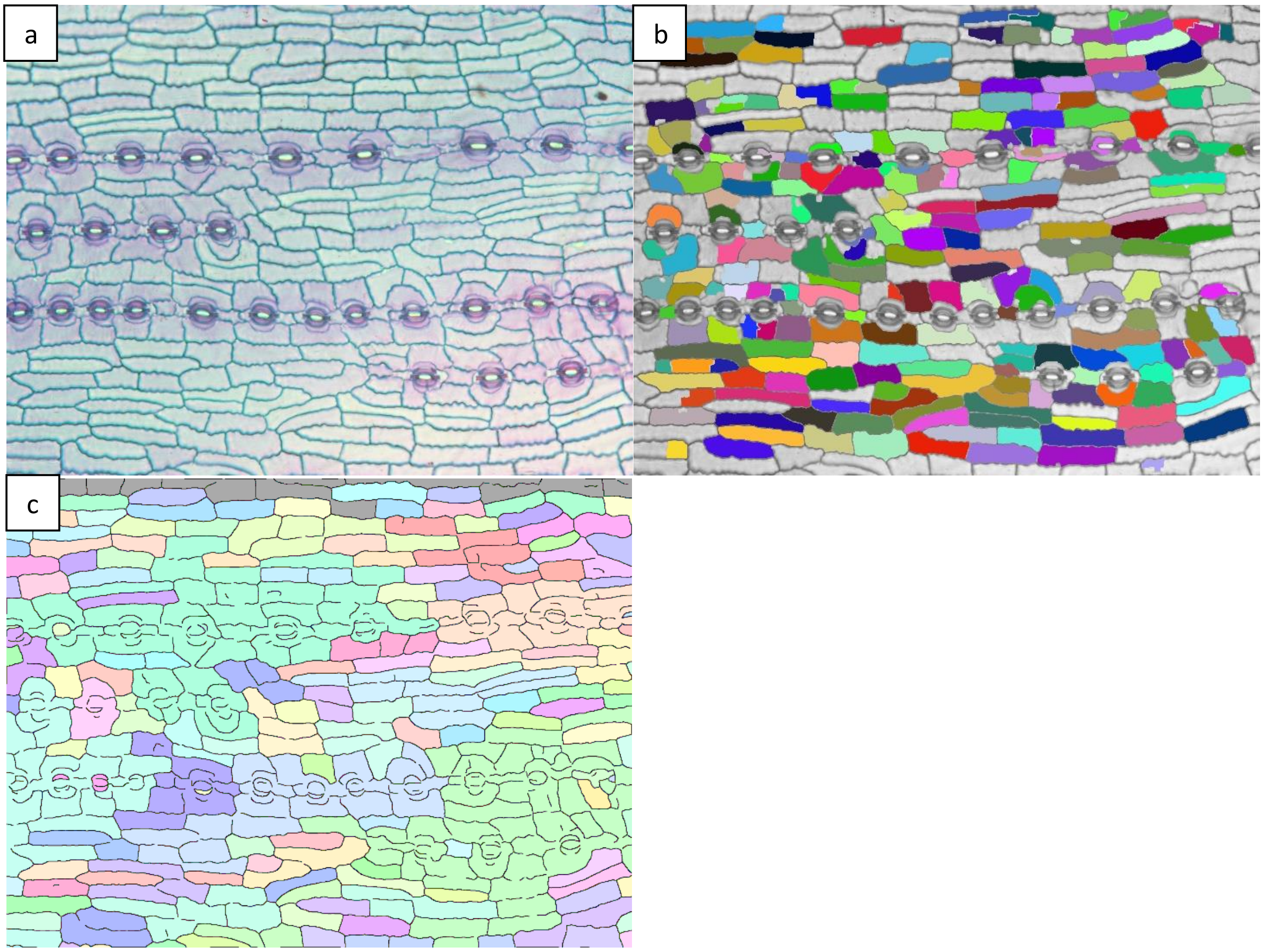


**Figure S8.** Automatic cell segmentation of a high-quality image of *Podocarpus coriaceus*. The raw image (a), PaCeQUant segmentation (b), segmentation after ridge detection (c).
